# The CBI‐R detects early behavioural impairment in genetic frontotemporal dementia

**DOI:** 10.1002/acn3.51544

**Published:** 2022-03-26

**Authors:** Annabel Nelson, Lucy L. Russell, Georgia Peakman, Rhian S. Convery, Arabella Bouzigues, Caroline V. Greaves, Martina Bocchetta, David M. Cash, John C. van Swieten, Lize Jiskoot, Fermin Moreno, Raquel Sanchez‐Valle, Robert Laforce, Caroline Graff, Mario Masellis, Maria Carmela Tartaglia, James B. Rowe, Barbara Borroni, Elizabeth Finger, Matthis Synofzik, Daniela Galimberti, Rik Vandenberghe, Alexandre de Mendonça, Chris R. Butler, Alexander Gerhard, Simon Ducharme, Isabelle Le Ber, Isabel Santana, Florence Pasquier, Johannes Levin, Markus Otto, Sandro Sorbi, Jonathan D. Rohrer, Genetic FTD Initiative (GENFI), Sónia Afonso, Maria Rosario Almeida, Sarah Anderl‐Straub, Christin Andersson, Anna Antonell, Silvana Archetti, Andrea Arighi, Mircea Balasa, Myriam Barandiaran, Nuria Bargalló, Robart Bartha, Benjamin Bender, Alberto Benussi, Maxime Bertoux, Anne Bertrand, Valentina Bessi, Sandra Black, Martina Bocchetta, Sergi Borrego‐Ecija, Jose Bras, Alexis Brice, Rose Bruffaerts, Agnès Camuzat, Marta Cañada, Valentina Cantoni, Paola Caroppo, David Cash, Miguel Castelo‐Branco, Olivier Colliot, Thomas Cope, Vincent Deramecourt, María de Arriba, Giuseppe Di Fede, Alina Díez, Diana Duro, Chiara Fenoglio, Camilla Ferrari, Catarina B. Ferreira, Nick Fox, Morris Freedman, Giorgio Fumagalli, Aurélie Funkiewiez, Alazne Gabilondo, Roberto Gasparotti, Serge Gauthier, Stefano Gazzina, Giorgio Giaccone, Ana Gorostidi, Caroline Greaves, Rita Guerreiro, Carolin Heller, Tobias Hoegen, Begoña Indakoetxea, Vesna Jelic, Hans‐Otto Karnath, Ron Keren, Gregory Kuchcinski, Tobias Langheinrich, Thibaud Lebouvier, Maria João Leitão, Albert Lladó, Gemma Lombardi, Sandra Loosli, Carolina Maruta, Simon Mead, Lieke Meeter, Gabriel Miltenberger, Rick van Minkelen, Sara Mitchell, Katrina Moore, Benedetta Nacmias, Annabel Nelson, Linn Öijerstedt, Jaume Olives, Sebastien Ourselin, Alessandro Padovani, Jessica Panman, Janne M. Papma, Yolande Pijnenburg, Cristina Polito, Enrico Premi, Sara Prioni, Catharina Prix, Rosa Rademakers, Veronica Redaelli, Daisy Rinaldi, Tim Rittman, Ekaterina Rogaeva, Adeline Rollin, Pedro Rosa‐Neto, Giacomina Rossi, Martin Rossor, Beatriz Santiago, Dario Saracino, Sabrina Sayah, Elio Scarpini, Sonja Schönecker, Harro Seelaar, Elisa Semler, Rachelle Shafei, Christen Shoesmith, Imogen Swift, Miguel Tábuas‐Pereira, Mikel Tainta, Ricardo Taipa, David Tang‐Wai, David L. Thomas, Paul Thompson, Hakan Thonberg, Carolyn Timberlake, Pietro Tiraboschi, Emily Todd, Philip Van Damme, Mathieu Vandenbulcke, Michele Veldsman, Jorge Villanua, Jason Warren, Carlo Wilke, Elisabeth Wlasich, Henrik Zetterberg, Miren Zulaica, Ana Verdelho

**Affiliations:** ^1^ Dementia Research Centre, Department of Neurodegenerative Disease UCL Queen Square Institute of Neurology London UK; ^2^ Department of Medical Statistics London School of Hygiene and Tropical Medicine London UK; ^3^ Department of Neurology Erasmus Medical Centre Rotterdam Netherlands; ^4^ Cognitive Disorders Unit, Department of Neurology Donostia Universitary Hospital San Sebastian Spain; ^5^ Neuroscience Area Biodonostia Health Research Institute San Sebastian Gipuzkoa Spain; ^6^ Alzheimer's Disease and Other Cognitive Disorders Unit, Neurology Service, Hospital Clínic, Institut d'Investigacións Biomèdiques August Pi I Sunyer University of Barcelona Barcelona Spain; ^7^ Clinique Interdisciplinaire de Mémoire, Département des Sciences Neurologiques, CHU de Québec, and Faculté de Médecine Université Laval Québec Canada; ^8^ Center for Alzheimer Research, Division of Neurogeriatrics, Department of Neurobiology, Care Sciences and Society Bioclinicum, Karolinska Institutet Solna Sweden; ^9^ Unit for Hereditary Dementias, Theme Aging Karolinska University Hospital Solna Sweden; ^10^ Sunnybrook Health Sciences Centre, Sunnybrook Research Institute University of Toronto Toronto Canada; ^11^ Tanz Centre for Research in Neurodegenerative Diseases University of Toronto Toronto Ontario Canada; ^12^ Department of Clinical Neurosciences University of Cambridge Cambridge UK; ^13^ Neurology Unit, Department of Clinical and Experimental Sciences University of Brescia Brescia Italy; ^14^ Department of Clinical Neurological Sciences University of Western Ontario London Ontario Canada; ^15^ Department of Neurodegenerative Diseases, Hertie‐Institute for Clinical Brain Research and Center of Neurology University of Tübingen Tübingen Germany; ^16^ Center for Neurodegenerative Diseases (DZNE Tübingen Germany; ^17^ Fondazione Ca’ Granda, IRCCS Ospedale Policlinico Milan Italy; ^18^ University of Milan, Centro Dino Ferrari Milan Italy; ^19^ Laboratory for Cognitive Neurology, Department of Neurosciences KU Leuven Leuven Belgium; ^20^ Neurology Service, University Hospitals Leuven Leuven Belgium; ^21^ Leuven Brain Institute, KU Leuven Leuven Belgium; ^22^ Faculty of Medicine, University of Lisbon Lisbon Portugal; ^23^ Nuffield Department of Clinical Neurosciences Medical Sciences Division, University of Oxford Oxford UK; ^24^ Department of Brain Sciences Imperial College London London UK; ^25^ Division of Neuroscience and Experimental Psychology, Wolfson Molecular Imaging Centre University of Manchester Manchester UK; ^26^ Departments of Geriatric Medicine and Nuclear Medicine University of Duisburg‐Essen Duisburg Germany; ^27^ Department of Psychiatry, McGill University Health Centre McGill University Montreal QC Canada; ^28^ McConnell Brain Imaging Centre, Montreal Neurological Institute McGill University Montreal QC Canada; ^29^ Sorbonne Université, Paris Brain Institute – Institut du Cerveau – ICM Inserm U1127, CNRS UMR 7225, AP‐HP – Hôpital Pitié‐Salpêtrière Paris France; ^30^ Département de Neurologie Centre de référence des démences rares ou précoces, IM2A, AP‐HP – Hôpital Pitié‐Salpêtrière Paris France; ^31^ Département de Neurologie AP‐HP – Hôpital Pitié‐Salpêtrière Paris France; ^32^ Reference Network for Rare Neurological Diseases (ERN‐RND), European Union; ^33^ University Hospital of Coimbra (HUC), Neurology Service, Faculty of Medicine University of Coimbra Coimbra Portugal; ^34^ Center for Neuroscience and Cell Biology, Faculty of Medicine University of Coimbra Coimbra Portugal; ^35^ Univ Lille Lille France; ^36^ Inserm 1172 Lille France; ^37^ CHU, CNR‐MAJ, Labex Distalz LiCEND Lille Lille France; ^38^ Department of Neurology Ludwig‐Maximilians Universität München Munich Germany; ^39^ German Center for Neurodegenerative Diseases (DZNE) Munich Germany; ^40^ Munich Cluster of Systems Neurology (SyNergy) Munich Germany; ^41^ Department of Neurology University of Ulm Ulm Germany; ^42^ Department of Neurofarba University of Florence Florence Italy; ^43^ IRCCS Fondazione Don Carlo Gnocchi Florence Italy

## Abstract

**Introduction:**

Behavioural dysfunction is a key feature of genetic frontotemporal dementia (FTD) but validated clinical scales measuring behaviour are lacking at present.

**Methods:**

We assessed behaviour using the revised version of the Cambridge Behavioural Inventory (CBI‐R) in 733 participants from the Genetic FTD Initiative study: 466 mutation carriers (195 *C9orf72*, 76 *MAPT*, 195 *GRN*) and 267 non‐mutation carriers (controls). All mutation carriers were stratified according to their global CDR plus NACC FTLD score into three groups: asymptomatic (CDR = 0), prodromal (CDR = 0.5) and symptomatic (CDR = 1+). Mixed‐effects models adjusted for age, education, sex and family clustering were used to compare between the groups. Neuroanatomical correlates of the individual domains were assessed within each genetic group.

**Results:**

CBI‐R total scores were significantly higher in all CDR 1+ mutation carrier groups compared with controls [*C9orf72* mean 70.5 (standard deviation 27.8), *GRN* 56.2 (33.5), *MAPT* 62.1 (36.9)] as well as their respective CDR 0.5 groups [*C9orf72* 13.5 (14.4), *GRN* 13.3 (13.5), *MAPT* 9.4 (10.4)] and CDR 0 groups [*C9orf72* 6.0 (7.9), *GRN* 3.6 (6.0), *MAPT* 8.5 (13.3)]. The *C9orf72* and *GRN* 0.5 groups scored significantly higher than the controls. The greatest impairment was seen in the Motivation domain for the *C9orf72* and *GRN* symptomatic groups, whilst in the symptomatic *MAPT*group, the highest‐scoring domains were Stereotypic and Motor Behaviours and Memory and Orientation. Neural correlates of each CBI‐R domain largely overlapped across the different mutation carrier groups.

**Conclusions:**

The CBI‐R detects early behavioural change in genetic FTD, suggesting that it could be a useful measure within future clinical trials.

## Introduction

Frontotemporal dementia (FTD) is a heterogeneous neurodegenerative disease associated with changes in behaviour, language and cognition.^1^ Around one‐third of FTD is autosomal dominant^2^ with the main genetic causes being mutations in microtubule‐associated protein tau (*MAPT*),^3^ progranulin (*GRN*)^4^ and chromosome 9 open reading frame 72 (*C9orf72*).^5^ Most commonly, familial FTD will present with changes in personality and social conduct, known as behavioural variant FTD (bvFTD). However, despite the development of a number of therapeutic drugs for genetic FTD and trials now starting, few validated scales have been developed to detect and monitor the underlying behavioural changes in FTD. Such measures will be important in assessing the potential effectiveness of disease‐modifying therapies.

Unlike a number of already existing scales such as the Neuropsychiatric Inventory (NPI)^6^ which do not encompass all the core diagnostic features of FTD, the Cambridge Behavioural Inventory was specifically designed to focus on the changes seen in those with bvFTD.^7^ The revised version (CBI‐R) is a 45‐item questionnaire measuring the severity of symptoms across 10 domains. Four of the domains encompass the core behavioural criteria in the diagnosis of bvFTD^1^: Motivation (including both apathy and loss of empathy), Stereotypic and Motor Behaviours (i.e. obsessive–compulsive behaviour), Eating Habits (such as preference for sweet foods) and Abnormal Behaviour (including disinhibition and impulsivity). Three further domains cover neuropsychiatric symptoms that occur regularly in FTD, particularly in the genetic forms: Beliefs (delusions and hallucinations), Mood (depression, agitation and irritability) and Sleep (increased daytime or disturbed sleep). Two domains focus on functional deficits: Everyday Skills (such as difficulties with handling money and using items around the house) and Self Care. The last domain is Memory and Orientation which includes deficits seen in FTD such as impaired attention and concentration. However, although the CBI‐R has been investigated across a number of phenotypic variants of FTD,^8^, ^9^, ^10^ little work has been done to understand how well it measures the behavioural and functional change in genetic forms of FTD, particularly in the presymptomatic period. Furthermore, few studies have been performed to examine the neural correlates of the CBI‐R.^9^

The aim of this study was therefore to assess the CBI‐R as a measure of behavioural and functional change in genetic FTD using data from the Genetic FTD Initiative (GENFI) cohort, an international study of the natural history of genetic FTD with the aim of identifying early biomarkers.

## Methods

### Participants

Data were collected from participants from the fifth data freeze of the GENFI study including sites in the UK, Canada, Sweden, Netherlands, Belgium, Spain, Portugal, Italy and Germany. Participants were recruited from families with a confirmed pathogenic genetic mutation in *GRN, MAPT* or *C9orf72,* including those who were symptomatic as well as those who were at‐risk first‐degree relatives of mutation carriers. This second group consists of both presymptomatic mutation carriers and mutation‐negative family members who therefore act as healthy controls. Of the 849 participants recruited into the fifth data freeze, cross‐sectional data on the CBI‐R was available from 733 participants: 466 mutation carriers (195 *C9orf72,* 195 *GRN,* 76 *MAPT*) and 267 mutation‐negative healthy controls. The study procedures were approved by local ethics committees and all participants provided informed written consent.

### Procedure

All participants underwent the standardised GENFI clinical assessment battery including history and examination, the Mini‐Mental State Examination (MMSE), the Frontotemporal dementia Rating Scale (FRS) and the Clinical Dementia Rating Scale plus National Alzheimer Coordinating Centre FTLD module (CDR plus NACC FTLD). A global CDR plus NACC FTLD score gives a summary of the current disease stage, where 0 is asymptomatic, 0.5 is prodromal or very mildly symptomatic and 1, 2 and 3 represent the mild, moderate and severe fully symptomatic stages. For the purposes of this study, the fully symptomatic mutation carriers were grouped together as 1+. The CDR plus NACC FTLD sum of boxes score in which the total score on each domain is added together (max score = 24) provides a measure of disease severity.^11^, ^12^

### The revised version of the Cambridge Behavioural Inventory

All participants had a CBI‐R questionnaire completed by a close informant, usually either a family member or a close friend. The CBI‐R assesses the frequency of the given behaviour over the past month on a scale of 0–4^7^: a score of 0 means that there is no impairment, a score of 1 an occasional occurrence (a few times a month), 2 a repeated occurrence (few times a week), 3 a daily occurrence and 4 is a constant occurrence. Higher scores, therefore, represent more severe behavioural or functional deficits. There are 45 items in total, meaning that the CBI‐R total score has a maximum of 180. Each domain contains between two and eight items, and therefore, the maximum domain score can vary from 8 to 32. For the purposes of comparing the different behaviours, we converted individual domain scores into percentages of the total maximum score.

### MRI

Participants underwent volumetric T1‐weighted magnetic resonance imaging according to the harmonised GENFI protocol on a 3T scanner. This usually occurred on the same day as the clinical assessment but occasionally on a different day at a maximum of 12 weeks apart. All images were checked for quality control, and scans with movement or artefacts were removed from the analysis. Only scans from mutation carriers were included in the correlative analysis: of the 466 participants, 430 scans were available for the analysis: 179 *C9orf72*, 182 *GRN* and 69 *MAPT* mutation carriers.

Neuroanatomical regions of interest were subsequently generated as previously described using an automated atlas segmentation propagation and label fusion strategy called Geodesic Information Flow.^13^ Specifically, volumes of the frontal, temporal, insular and parietal cortices as well as hippocampus, amygdala, thalamus and striatum were calculated and expressed as a percentage of total intracranial volume, computed with SPM12 (Statistical Parametric Mapping, Welcome Trust Centre for Neuroimaging, London, UK) running under Matlab R2014b.^14^

### Statistical analysis

All statistical analyses were performed using StataCorp. 2016. Stata Statistical Software: Version 14. College Station, TV: StataCorp LLC.

Demographic data were compared between groups using a linear regression model, with bootstrapping when the data were not normally distributed.

In the healthy control group, Spearman's rank correlations were performed to evaluate the relationship between the CBI‐R total score and both age and education. To assess the relationship of CBI‐R total score with sex, a Mann–Whitney *U* test was used.

In order to compare the CBI‐R total score between groups, a mixed‐effects model was used that adjusted for age, education, sex and family clustering, along with bootstrapped confidence intervals with 2000 repetitions as the data were not normally distributed.

The relationship of the CBI‐R total score with disease severity (both CDR plus NACC FTLD sum of boxes and FRS) was assessed using Spearman’s rank correlations within each genetic group.

Similar mixed‐effects models as performed above were used to assess differences in each of the individual CBI‐R domains firstly, between genetic groups, and secondly, within groups (symptomatic mutation carriers only).

Neural correlates of each individual CBI‐R domain were investigated in each genetic group by assessing non‐parametric partial correlations (adjusting for age and disease severity) of the domain score with the neuroanatomical regions of interest.

## Results

### Demographics

The age, sex and education of the participants are shown in Table 1. All symptomatic mutation carriers and the prodromal *GRN* mutation carriers were significantly older than controls (all *p* < 0.050) while the asymptomatic *MAPT* mutation carriers were younger than controls (*p* < 0.001). Within each of the genetic groups, the symptomatic mutation carriers were significantly older than the prodromal and asymptomatic mutation carriers (all *p* < 0.050). The symptomatic *C9orf72* group contained more males than controls (*p* < 0.001) and compared with their asymptomatic and prodromal groups (*p* = 0.002 and *p* = 0.019 respectively), but there were no other differences in sex compared with controls or between genetic groups. Symptomatic *C9orf72* and *GRN* mutation carriers spent significantly fewer years in education than controls (*p* = 0.004 and *p* < 0.001 respectively) and their respective asymptomatic groups (*C9orf72*, *p* = 0.020; *GRN*, *p* < 0.001), and for the *GRN* group also less than their prodromal mutation carriers (*p* = 0.011).

**Table 1:** Demographics and CBI‐R scores (total and individual domains) for healthy controls and each genetic group split by global CDR plus NACC FTLD score (0 = asymptomatic, 0.5 = prodromal, 1+ = symptomatic). *N* represents the number of participants.

	Healthy controls	C9orf72			GRN			MAPT		
		0	0.5	1+	0	0.5	1+	0	0.5	1+
*N*	267	94	34	67	122	26	47	42	13	21
Sex	41%	42%	41%	66%	33%	46%	47%	41%	31%	57%
Age (years)	46.4 (13.0)	43.9 (11.6)	49.7 (11.2)	62.6 (9.4)	45.8 (12.1)	52.1 (13.7)	63.0 (7.4)	38.6 (11.0)	46.4 (12.8)	58.9 (9.4)
Education (years)	14.4 (3.4)	14.3 (3.0)	13.9 (2.6)	13.0 (3.8)	14.7 (3.5)	13.8 (4.2)	11.7 (3.4)	14.4 (3.4)	13.6 (2.5)	13.6 (4.0)
MMSE (/30)	29.4 (1.2)	29.1 (1.2)	28.4 (2.2)	23.3 (6.7)	29.5 (0.8)	28.4 (2.6)	20.1 (7.7)	29.5 (0.8)	28.1 (2.3)	21.9 (8.1)
Motivation (/20)	0.5 (1.6)	0.8 (2.1)	1.9 (3.8)	10.5 (6.1)	0.2 (0.8)	2.2 (3.7)	10.0 (6.3)	1.4 (3.1)	1.2 (2.4)	9.2 (6.6)
Stereotypic and Motor Behaviours (/16)	0.5 (1.3)	0.7 (1.5)	1.6 (2.2)	6.6 (4.5)	0.4 (1.1)	1.5 (2.3)	3.8 (3.8)	1.2 (2.5)	1.0 (1.4)	7.6 (4.5)
Eating Habits (/16)	0.3 (0.8)	0.3 (0.9)	0.9 (2.4)	6.6 (4.9)	0.1 (0.5)	1.0 (2.4)	5.2 (4.5)	0.5 (1.6)	0.5 (1.2)	6.6 (5.5)
Abnormal Behaviour (/24)	0.6 (1.5)	0.9 (1.7)	1.8 (2.7)	7.9 (5.4)	0.5 (1.0)	1.4 (2.4)	5.8 (5.0)	1.0 (1.9)	1.2 (1.6)	7.2 (6.0)
Beliefs (/12)	0.0 (0.2)	0.0 (0.0)	0.1 (0.4)	1.5 (2.4)	0.0 (0.1)	0.0 (0.0)	1.0 (2.2)	0.0 (0.0)	0.1 (0.3)	0.5 (0.8)
Mood (/16)	1.1 (1.8)	1.1 (1.6)	1.2 (2.1)	4.2 (3.1)	0.8 (1.5)	1.7 (1.5)	3.9 (3.3)	2.0 (2.7)	1.5 (1.7)	3.9 (3.2)
Sleep (/8)	0.6 (1.2)	0.6 (1.3)	1.2 (1.8)	3.0 (2.1)	0.4 (0.9)	1.0 (1.3)	2.9 (2.4)	1.1 (1.5)	0.5 (0.8)	1.7 (1.4)
Everyday Skills (/20)	0.2 (0.9)	0.1 (0.6)	0.6 (1.5)	8.9 (5.8)	0.1 (0.6)	0.5 (1.2)	7.1 (6.6)	0.2 (0.7)	0.2 (0.4)	6.8 (6.7)
Self Care (/16)	0.0 (0.4)	0.2 (0.9)	0.9 (2.5)	5.3 (5.3)	0.0 (0.1)	0.1 (0.4)	2.7 (4.6)	0.2 (1.2)	0.1 (0.3)	2.4 (4.6)
Memory and Orientation (/32)	1.3 (2.3)	1.3 (2.4)	2.9 (3.8)	16.0 (6.6)	1.0 (2.2)	3.9 (4.3)	13.6 (8.4)	1.3 (2.1)	3.1 (3.7)	16.2 (8.6)
CBI‐R Total Score (/180)	5.2 (7.8)	6.0 (7.9)	13.5 (14.4)	70.5 (27.8)	3.6 (6.0)	13.3 (13.5)	56.2 (33.5)	8.9 (13.3)	9.4 (10.4)	62.1 (36.9)

Sex is shown as the percentage of males in the group. All other scores are shown as mean (standard deviation). MMSE, Mini‐Mental State Examination.

### CBI‐R in healthy controls

Healthy controls (i.e. mutation‐negative family members) had very low scores on the CBI‐R with the highest scoring domain being Memory and Orientation [mean (standard deviation) 1.3 (2.3)] and Mood [1.1 (1.6)]. The mean CBI‐R total score was only 5.2 (standard deviation 7.8) out of 180. There were no significant correlations of CBI‐R total score with either age (*r* = 0.06, *p* = 0.310) or education (*r* = − 0.06, *p* = 0.358), and there was no difference in score between men and women (*p* = 0.435).

### CBI‐R total score

**Figure 1:**
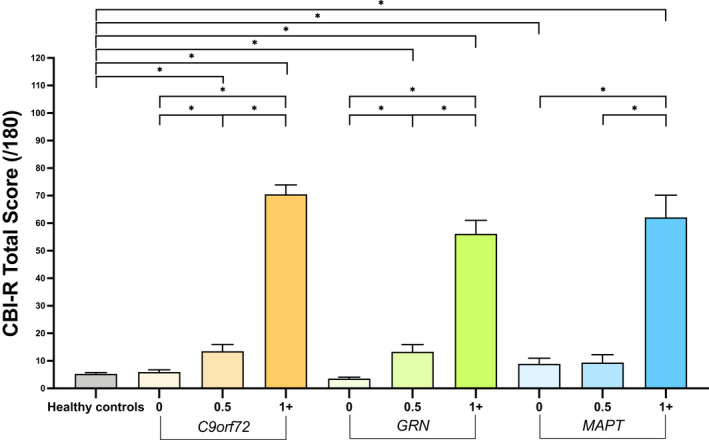
Mean CBI‐R total scores in healthy controls and each genetic group stratified by global CDR plus NACC FTLD scores. Significant differences between controls and within each genetic group are starred. Differences between different genetic groups are not shown. Error bars represent the standard error of the mean.

All symptomatic mutation carrier groups scored significantly higher than controls (Fig. 1, Table 1, Table S1): *C9orf72* 70.5 (27.8), *GRN* 56.2 (33.5), *MAPT* 62.1 (36.9). In the prodromal groups, the *C9orf72* and *GRN* mutation carriers scored significantly higher than controls [*C9orf72* 13.5 (14.4), *GRN* 13.3 (13.5)] with only a trend for a higher score in the *MAPT* mutation carriers: 9.4 (10.4). In the asymptomatic mutation carriers, there was no difference between the *C9orf72* [6.0 (7.9) or *GRN* 3.6 (6.0)] and controls but there was a significantly higher score in the *MAPT* mutation carriers: 8.9 (13.3).

Comparing *within* groups, the symptomatic mutation carriers all scored higher than their respective prodromal mutation carriers and asymptomatic mutation carriers in each genetic group (Fig. 1, Table 1, Table S1). Prodromal *C9orf72* and *GRN* mutation carriers also scored higher than their asymptomatic mutation carriers.

Comparing *between* groups of the same global CDR plus NACC FTLD stage, symptomatic *C9orf72* mutation carriers scored significantly higher than symptomatic *GRN* mutation carriers but there were no other differences between the symptomatic mutation carrier groups (Table S1). There were also no differences between the prodromal mutation carriers. In the asymptomatic mutation carriers, the *MAPT* group scored significantly higher than the *GRN* group but there were no other differences.

**Figure 2:**
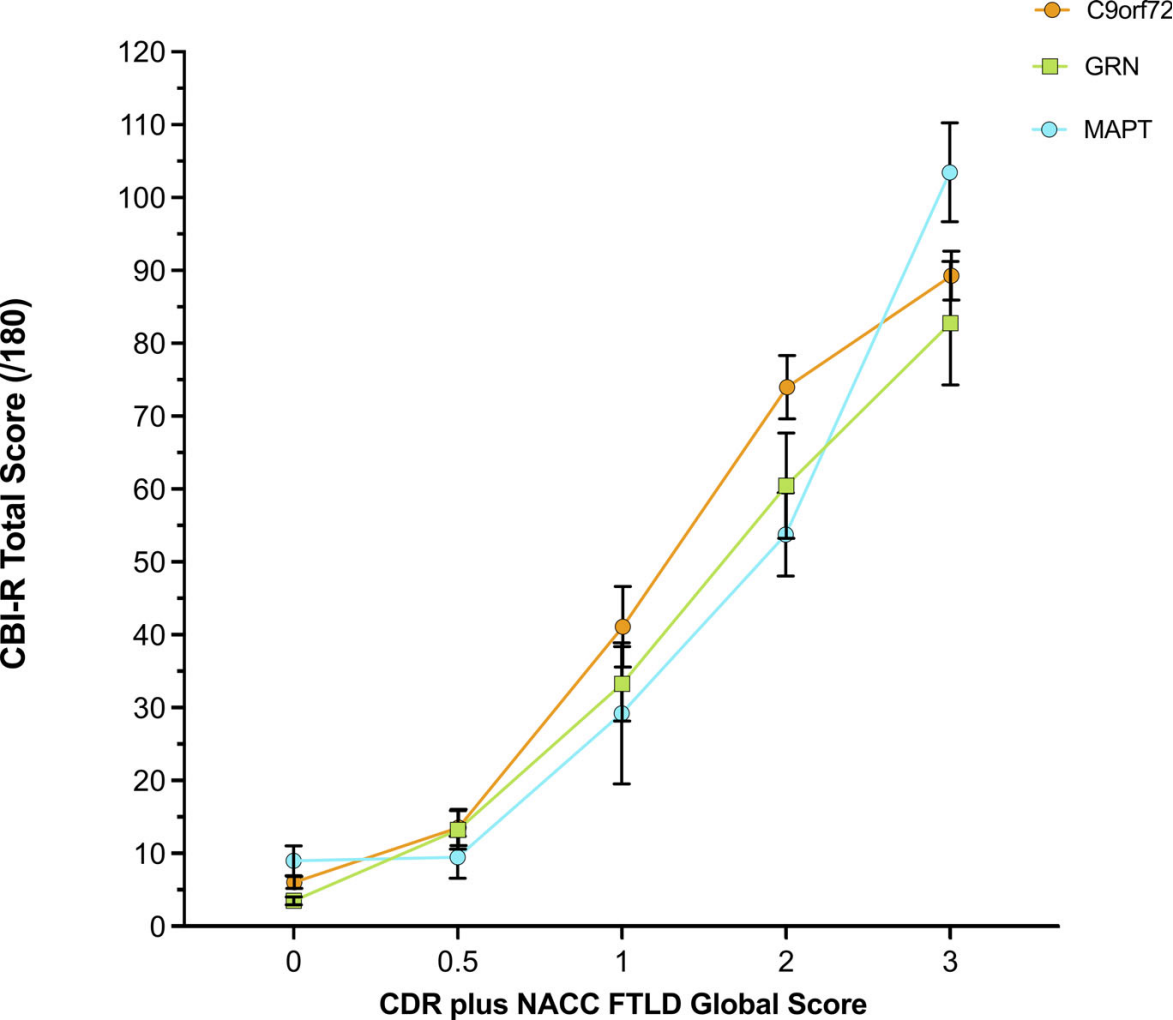
Cross‐sectional CBI‐R total scores (mean with standard errors) in each genetic group by CDR plus NACC FTLD global score.

Figure 2 shows the CBI‐R total score in each of the genetic groups when the fully symptomatic group is stratified into individual CDR plus NACC FTLD stages of mild (1), moderate (2) and severe (3).

### Comparison of CBI‐R total score with measures of disease severity

There was a significant positive correlation between the CBI‐R total score and the CDR plus NACC FTLD sum of boxes in all mutation carrier groups (*C9orf72 r* = 0.78, *GRN r* = 0.82, *MAPT r* = 0.60, all *p* = <0.001) (Fig. S1).

A significant negative correlation was seen in each mutation carrier group between CBI‐R total scores and FRS (*C9orf72 r* = −0.92, *GRN r* = −0.88, *MAPT r* = −0.88, all *p* = <0.001) (Fig. S2).

### Individual CBI‐R domain scores

**Figure 3:**
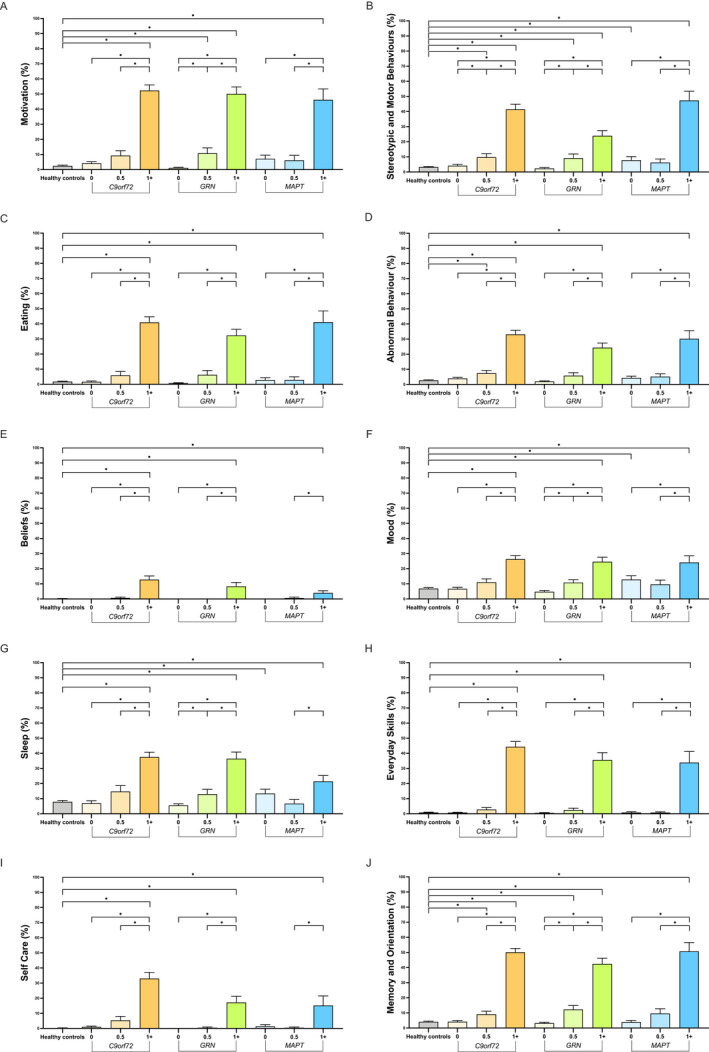
Mean CBI‐R scores (expressed as a percentage to allow comparison) in each of the individual 10 domains within healthy controls and each genetic group stratified by CDR plus NACC FTLD global scores. Significant differences between controls and within each genetic group are starred. Differences between different genetic groups are not shown. Error bars represent the standard error of the mean.

Looking at each domain individually (Fig. 3, Table S2), all symptomatic mutation carrier groups scored significantly higher than controls in every domain. In the prodromal mutation carriers, the *C9orf72* group scored higher than controls in Stereotypic and Motor Behaviours, Abnormal Behaviour and Memory and Orientation, whilst the *GRN* group scored higher than controls in Motivation, Stereotypic and Motor Behaviours and Memory and Orientation. The prodromal *MAPT* group did not score significantly different to controls in any of the domains but the asymptomatic *MAPT* group scored higher than controls in Stereotypic and Motor Behaviours, Sleep and Mood. Neither asymptomatic *C9orf72* or *GRN* groups scored higher in any domains compared with controls.

*Within* group differences are shown in Figure 3 and Table S2. For *C9orf72* and *GRN* mutation carriers, the symptomatic mutation carriers scored higher in all the domains compared with their respective prodromal and asymptomatic mutation carriers. This was similar for *MAPT* mutation carriers in most domains except Beliefs, Sleep and Self Care, where the symptomatic mutation carriers scored higher than the prodromal but not the asymptomatic mutation carriers. The *C9orf72* prodromal group scored higher than the asymptomatic group in Stereotypic and Motor Behaviours, whereas the *GRN* prodromal group scored higher than the asymptomatic group in Motivation, Stereotypic and Motor Behaviours, Abnormal Behaviour, Mood, Sleep and Memory and Orientation. The prodromal *MAPT* group did not score significantly different to their asymptomatic group in any of the domains.

Comparing *between* groups of the same global CDR plus NACC FTLD stage, symptomatic *C9orf72* mutation carriers scored higher than *GRN* mutation carriers for Stereotypic and Motor Behaviours and Abnormal Behaviour and Self Care and higher than *MAPT* mutation carriers for Beliefs and Sleep (Table S2). Symptomatic *MAPT* mutation carriers scored higher than *GRN* mutation carriers for Stereotypic and Motor Behaviours and vice versa for Sleep. Although there were no differences between the prodromal groups, in the asymptomatic groups, the *MAPT* mutation carriers scored higher than the *GRN* mutation carriers for Motivation, Stereotypic and Motor Behaviours, Mood and Sleep and higher than the *C9orf72* mutation carriers for Sleep.

Comparing *between domains* in each of the symptomatic mutation carrier groups (Fig. S2, Table S3), the highest‐scoring domain was Motivation in both *C9orf72* mutation carriers (significantly higher than the other domains apart from Everyday Skills and Memory and Orientation) and *GRN* mutation carriers (significantly higher than all the other domains), whilst in the *MAPT* mutation carriers, it was Memory and Orientation (significantly higher than the other domains except Motivation, Stereotypic and Motor Behaviours and Eating Habits) followed by Stereotypic and Motor Behaviours (significantly higher than other domains except Motivation, Eating Habits and Memory and Orientation).

### Neural correlates of individual CBI‐R domains

Partial correlations between scores in each of the 10 domains and the neuroanatomical regions of interest adjusting for age and disease severity for each genetic group are shown in Tables S4–S6. There were no significant correlations with the Motivation domain. However, the Stereotypic and Motor Behaviours domain score negatively correlated with the hippocampal and amygdala volume (particularly on the right) in the *C9orf72* and *GRN* mutation carriers. Eating Habits score negatively correlated particularly with insula volume (bilaterally) in the *C9orf72* and *GRN* mutation carriers and with frontal lobe volumes to a lesser extent in all three groups. The Abnormal Behaviour domain scores negatively correlated with hippocampal volume (right more than left) in the *C9orf72* and *GRN* mutation carriers and with left insula volume in the *MAPT* mutation carriers. Beliefs score negatively correlated with thalamus volume in both the *C9orf72* and *GRN* mutation carriers but also the frontal and temporal lobes and striatum in the *C9orf72* group. Mood and Sleep both negatively correlated with medial temporal lobe volumes in the *GRN* group, with Mood showing a similar correlation in the *C9orf72* mutation carriers. The Everyday Skills domain score negatively correlated with frontal and temporal lobe volumes in *C9orf72* and *MAPT* mutation carriers, with the insula in *C9orf72* and *GRN* mutation carriers, and with the striatum in the *GRN* and *MAPT* groups. Self Care score negatively correlated with frontal, insula and parietal volumes in the *C9orf72* group, but quite widespread, mainly right‐sided, cortical and subcortical volumes in the *GRN* group. Lastly, Memory and Orientation negatively correlated with hippocampal volume in all three groups and additionally with the thalamus in the *C9orf72* mutation carriers.

## Discussion

In this study, we have shown that the CBI‐R detects early behavioural change in familial FTD, with overlapping but distinct patterns of impairment in the three major genetic groups. The highest‐scoring domain was Motivation in the symptomatic *C9orf72* and *GRN* groups, and Stereotypic and Motor Behaviours and Memory and Orientation in the *MAPT* group. CBI‐R total score was positively correlated with CDR plus NACC FTLD sum of boxes score and negatively correlated with the FRS which suggest that progression of overall behavioural change as measured by the CBI‐R tracks with disease severity. Lastly, we found overlapping neural correlates of individual CBI‐R domains across the different genetic groups.

In symptomatic mutation carriers, we found that the CBI‐R can detect behavioural and functional differences across the range of domains included. In this study, we found that symptomatic *C9orf72* mutation carriers scored highest in the CBI‐R. This may relate in part to the particular presence of neuropsychiatric features such as delusions and hallucinations (here recorded as Beliefs) in *C9orf72* mutation carriers which occur more commonly in this group in addition to the core behavioural features common to all three genetic forms.^15^ However, symptomatic mutation carriers also scored higher on functional measures (e.g. Self Care) which in *C9orf72* mutation carriers can be multifactorial due to a combination of behavioural, cognitive and motor deficits.

In both symptomatic *C9orf72* and *GRN* groups, we found that Motivation was the highest scoring domain. This domain includes questions about both apathy and loss of empathy, both core behavioural features of FTD and both symptoms reported to be prominent in these two forms of genetic FTD.^16^, ^17^ In contrast, in the symptomatic *MAPT* group, Stereotypic and Motor Behaviours (i.e. obsessive–compulsive behaviour) was the most common behavioural domain impaired, a feature described in prior studies.^18^ Additional to this, however, the *MAPT* group also scored highly in the Memory and Orientation domain, consistent with prior studies showing early episodic memory impairment in this subgroup.^19^

Behavioural changes have been reported presymptomatically in genetic FTD in a number of previous studies.^19^, ^20^, ^21^, ^22^ Usually, these changes occur late in the presymptomatic period as seen here, where impairment can be detected in prodromal mutation carriers, significantly so in the *GRN* and *C9orf72* mutation carriers in our study. By this time, structural and functional brain changes have occurred,^23^, ^24^, ^25^, ^26^ and cognitive disturbances also co‐occur.^27^, ^28^, ^29^ Interestingly, *MAPT* mutation carriers in the prodromal stage scored slightly lower than the *C9orf72* and *GRN* groups, with only a trend to a higher score than controls. However, *MAPT* mutation carriers in the asymptomatic period scored at a similar level to the other groups, but here were significantly more impaired than controls. The domains significantly affected in this asymptomatic *MAPT* group were Stereotypic Behaviours (i.e. the most frequent behavioural symptom during the symptomatic period) as well as Mood and Sleep. Potentially, the CBI‐R can therefore pick up the very early behavioural change in genetic FTD, here detecting symptoms many years before likely onset.

Neural correlates overlapped across the different genetic groups, although with some differences. The Stereotypic and Motor Behaviours domain was associated with medial temporal lobe atrophy, consistent with prior studies showing that obsessive–compulsive behaviours in FTD correlate with hippocampal and amygdala volumes^30^ and are seen particularly in sporadic FTD in those with temporal variant FTD.^31^, ^32^ A change in eating was associated with insula volumes in some of the groups, and frontal lobe volumes in all groups, a finding previously shown in a number of previous studies (where the association is usually with orbitofrontal atrophy).^33^, ^34^ Interestingly, the Abnormal Behaviour domain correlated with medial temporal atrophy, particularly on the right in *C9orf72* and *GRN* mutation carriers. The occurrence of disinhibition has been associated mainly with (orbito)frontal lobe atrophy previously, but some studies have implicated the right medial temporal lobe, suggesting a disruption of normal reward processing.^35^ The Beliefs domain score was associated with thalamic atrophy in both the *C9orf72* and *GRN* groups. This association of hallucinations and delusions has been shown previously in *C9orf72* mutation carriers^36^ and has previously been thought to be particularly distinct for this genetic group. However, here we show a similar association in the *GRN* mutation carriers. For the cognitive domain of Memory and Orientation, there was an association with hippocampal volume in all three groups, consistent with the known role of the medial temporal lobe in episodic memory. However, there was an additional association with thalamus atrophy in the *C9orf72* mutation carriers, a region long associated with episodic memory function.^37^

A limitation of our study was the relatively small sample size of the cohort once it is stratified, particularly for the *MAPT* mutation carriers, for example the prodromal *MAPT* group was relatively small, and negative results here may relate to the small sample size. We have also focused here on cross‐sectional data, extrapolating to changes over time across individuals but future research examining how the CBI‐R changes longitudinally over time within an individual will be important. Lastly, as the CBI‐R was commonly completed by an informant from a family with genetic FTD, it may be that they were more alert to the presence of behavioural symptoms, particularly in those who were prodromal, leading to potentially higher CBI‐R scores than in those with sporadic FTD.

## Conclusion

As we move into clinical trials for genetic FTD, the need for outcome measures that are both easy to assess and not time‐consuming is required. There are still few validated assessment scales that focus on the behavioural and functional deficits prominent in people with FTD. The benefit of the CBI‐R is that it includes core behavioural, neuropsychiatric, functional and cognitive measures within the same scale. Our study suggests the CBI‐R can detect early behavioural change in genetic FTD, making it potentially a useful marker in a clinical trial setting. Measuring individual changes in behaviour over time will now be an important next step in understanding how the CBI‐R might be used in such trials.

## Conflict of Interest

None.

## Appendix

### GENFI Consortium Authors

• Sónia Afonso – Instituto Ciencias Nucleares Aplicadas a Saude, Universidade de Coimbra, Coimbra, Portugal;
• Maria Rosario Almeida – Faculty of Medicine, University of Coimbra, Coimbra, Portugal;
• Sarah Anderl‐Straub – Department of Neurology, University of Ulm, Ulm, Germany;
• Christin Andersson – Department of Clinical Neuroscience, Karolinska Institutet, Stockholm, Sweden;
• Anna Antonell – Alzheimer’s disease and Other Cognitive Disorders Unit, Neurology Service, Hospital Clínic, Barcelona, Spain;
• Silvana Archetti – Biotechnology Laboratory, Department of Diagnostics, ASST Brescia Hospital, Brescia, Italy;
• Andrea Arighi – Fondazione IRCCS Ca′ Granda Ospedale Maggiore Policlinico, Neurodegenerative Diseases Unit, Milan, Italy; University of Milan, Centro Dino Ferrari, Milan, Italy;
• Mircea Balasa – Alzheimer’s disease and Other Cognitive Disorders Unit, Neurology Service, Hospital Clínic, Barcelona, Spain;
• Myriam Barandiaran – Cognitive Disorders Unit, Department of Neurology, Donostia University Hospital, San Sebastian, Gipuzkoa, Spain; Neuroscience Area, Biodonostia Health Research Insitute, San Sebastian, Gipuzkoa, Spain;
• Nuria Bargalló – Imaging Diagnostic Center, Hospital Clínic, Barcelona, Spain;
• Robart Bartha – Department of Medical Biophysics, The University of Western Ontario, London, Ontario, Canada; Centre for Functional and Metabolic Mapping, Robarts Research Institute, The University of Western Ontario, London, Ontario, Canada;
• Benjamin Bender – Department of Diagnostic and Interventional Neuroradiology, University of Tübingen, Tübingen, Germany;
• Alberto Benussi – Centre for Neurodegenerative Disorders, Department of Clinical and Experimental Sciences, University of Brescia, Italy;
• Maxime Bertoux – Inserm 1172, Lille, France; CHU, CNR‐MAJ, Labex Distalz, LiCEND Lille, France;
• Anne Bertrand – Sorbonne Université, Paris Brain Institute – Institut du Cerveau – ICM, Inserm U1127, CNRS UMR 7225, AP‐HP – Hôpital Pitié‐Salpêtrière, Paris, France; Inria, Aramis project‐team, F‐75013, Paris, France; Centre pour l'Acquisition et le Traitement des Images, Institut du Cerveau et la Moelle, Paris, France
• Valentina Bessi – Department of Neuroscience, Psychology, Drug Research, and Child Health, University of Florence, Florence, Italy;
• Sandra Black – Sunnybrook Health Sciences Centre, Sunnybrook Research Institute, University of Toronto, Toronto, Canada;
• Martina Bocchetta – Dementia Research Centre, Department of Neurodegenerative Disease, UCL Institute of Neurology, Queen Square, London, UK;
• Sergi Borrego‐Ecija – Alzheimer’s disease and Other Cognitive Disorders Unit, Neurology Service, Hospital Clínic, Barcelona, Spain;
• Jose Bras – Center for Neurodegenerative Science, Van Andel Institute, Grand Rapids, Michigan, MI 49503, USA;
• Alexis Brice – Sorbonne Université, Paris Brain Institute – Institut du Cerveau – ICM, Inserm U1127, CNRS UMR 7225, AP‐HP – Hôpital Pitié‐Salpêtrière, Paris, France; Reference Network for Rare Neurological Diseases (ERN‐RND);
• Rose Bruffaerts – Laboratory for Cognitive Neurology, Department of Neurosciences, KU Leuven, Leuven, Belgium;
• Agnès Camuzat – Sorbonne Université, Paris Brain Institute – Institut du Cerveau – ICM, Inserm U1127, CNRS UMR 7225, AP‐HP – Hôpital Pitié‐Salpêtrière, Paris, France;
• Marta Cañada – CITA Alzheimer, San Sebastian, Gipuzkoa, Spain
• Valentina Cantoni – Centre for Neurodegenerative Disorders, Neurology Unit, Department of Clinical and Experimental Sciences, University of Brescia, Brescia, Italy;
• Paola Caroppo – Fondazione IRCCS Istituto Neurologico Carlo Besta, Milano, Italy;
• David Cash – Dementia Research Centre, Department of Neurodegenerative Disease, UCL Institute of Neurology, Queen Square, London, UK;
• Miguel Castelo‐Branco – Faculty of Medicine, University of Coimbra, Coimbra, Portugal;
• Olivier Colliot – Sorbonne Université, Paris Brain Institute – Institut du Cerveau – ICM, Inserm U1127, CNRS UMR 7225, AP‐HP – Hôpital Pitié‐Salpêtrière, Paris, France; Inria, Aramis project‐team, F‐75013, Paris, France; Centre pour l'Acquisition et le Traitement des Images, Institut du Cerveau et la Moelle, Paris, France;
• Thomas Cope – Department of Clinical Neuroscience, University of Cambridge, Cambridge, UK;
• Vincent Deramecourt – Univ Lille, France; Inserm 1172, Lille, France; CHU, CNR‐MAJ, Labex Distalz, LiCEND Lille, France;
• María de Arriba – Neuroscience Area, Biodonostia Health Research Insitute, San Sebastian, Gipuzkoa, Spain;
• Giuseppe Di Fede – Fondazione IRCCS Istituto Neurologico Carlo Besta, Milano, Italy;
• Alina Díez – Neuroscience Area, Biodonostia Health Research Insitute, San Sebastian, Gipuzkoa, Spain
• Diana Duro – Faculty of Medicine, University of Coimbra, Coimbra, Portugal;
• Chiara Fenoglio – Fondazione IRCCS Ca′ Granda Ospedale Maggiore Policlinico, Neurodegenerative Diseases Unit, Milan, Italy; University of Milan, Centro Dino Ferrari, Milan, Italy;
• Camilla Ferrari – Department of Neuroscience, Psychology, Drug Research, and Child Health, University of Florence, Florence, Italy;
• Catarina B. Ferreira ‐Laboratory of Neurosciences, Institute of Molecular Medicine, Faculty of Medicine, University of Lisbon, Lisbon, Portugal;
• Nick Fox – Dementia Research Centre, Department of Neurodegenerative Disease, UCL Institute of Neurology, Queen Square, London, UK;
• Morris Freedman – Baycrest Health Sciences, Rotman Research Institute, University of Toronto, Toronto, Canada;
• Giorgio Fumagalli – Fondazione IRCCS Ca′ Granda Ospedale Maggiore Policlinico, Neurodegenerative Diseases Unit, Milan, Italy; University of Milan, Centro Dino Ferrari, Milan, Italy;
• Aurélie Funkiewiez – Centre de référence des démences rares ou précoces, IM2A, Département de Neurologie, AP‐HP – Hôpital Pitié‐Salpêtrière, Paris, France; Sorbonne Université, Paris Brain Institute – Institut du Cerveau – ICM, Inserm U1127, CNRS UMR 7225, AP‐HP – Hôpital Pitié‐Salpêtrière, Paris, France;
• Alazne Gabilondo ‐Neuroscience Area, Biodonostia Health Research Insitute, San Sebastian, Gipuzkoa, Spain;
• Roberto Gasparotti – Neuroradiology Unit, University of Brescia, Brescia, Italy
• Serge Gauthier – Alzheimer Disease Research Unit, McGill Centre for Studies in Ageing, Department of Neurology & Neurosurgery, McGill University, Montreal, Québec, Canada;
• Stefano Gazzina – Neurology, ASST Brescia Hospital, Brescia, Italy
• Giorgio Giaccone – Fondazione IRCCS Istituto Neurologico Carlo Besta, Milano, Italy;
• Ana Gorostidi – Neuroscience Area, Biodonostia Health Research Insitute, San Sebastian, Gipuzkoa, Spain;
• Caroline Greaves – Dementia Research Centre, Department of Neurodegenerative Disease, UCL Institute of Neurology, Queen Square, London, UK;
• Rita Guerreiro – Center for Neurodegenerative Science, Van Andel Institute, Grand Rapids, Michigan, MI 49503, USA;
• Carolin Heller – Dementia Research Centre, Department of Neurodegenerative Disease, UCL Institute of Neurology, Queen Square, London, UK;
• Tobias Hoegen – Neurologische Klinik, Ludwig‐Maximilians‐Universität München, Munich, Germany;
• Begoña Indakoetxea – Cognitive Disorders Unit, Department of Neurology, Donostia University Hospital, San Sebastian, Gipuzkoa, Spain; Neuroscience Area, Biodonostia Health Research Insitute, San Sebastian, Gipuzkoa, Spain;
• Vesna Jelic – Division of Clinical Geriatrics, Karolinska Institutet, Stockholm, Sweden;
• Hans‐Otto Karnath – Division of Neuropsychology, Hertie‐Institute for Clinical Brain Research and Center of Neurology, University of Tübingen, Tübingen, Germany;
• Ron Keren ‐The University Health Network, Toronto Rehabilitation Institute, Toronto, Canada;
• Gregory Kuchcinski – Univ Lille, France; Inserm 1172, Lille, France; CHU, CNR‐MAJ, Labex Distalz, LiCEND Lille, France;
• Tobias Langheinrich – Division of Neuroscience and Experimental Psychology, Wolfson Molecular Imaging Centre, University of Manchester, Manchester, UK; Manchester Centre for Clinical Neurosciences, Department of Neurology, Salford Royal NHS Foundation Trust, Manchester, UK;
• Thibaud Lebouvier – Univ Lille, France; Inserm 1172, Lille, France; CHU, CNR‐MAJ, Labex Distalz, LiCEND Lille, France;
• Maria João Leitão – Centre of Neurosciences and Cell Biology, Universidade de Coimbra, Coimbra, Portugal;
• Albert Lladó – Alzheimer's disease and Other Cognitive Disorders Unit, Neurology Service, Hospital Clínic, Barcelona, Spain;
• Gemma Lombardi – Department of Neuroscience, Psychology, Drug Research and Child Health, University of Florence, Florence, Italy;
• Sandra Loosli ‐Neurologische Klinik, Ludwig‐Maximilians‐Universität München, Munich, Germany;
• Carolina Maruta – Laboratory of Language Research, Centro de Estudos Egas Moniz, Faculty of Medicine, University of Lisbon, Lisbon, Portugal;
• Simon Mead – MRC Prion Unit, Department of Neurodegenerative Disease, UCL Institute of Neurology, Queen Square, London, UK;
• Lieke Meeter – Department of Neurology, Erasmus Medical Center, Rotterdam, Netherlands;
• Gabriel Miltenberger – Faculty of Medicine, University of Lisbon, Lisbon, Portugal;
• Rick van Minkelen – Department of Clinical Genetics, Erasmus Medical Center, Rotterdam, Netherlands;
• Sara Mitchell – Sunnybrook Health Sciences Centre, Sunnybrook Research Institute, University of Toronto, Toronto, Canada;
• Katrina Moore – Dementia Research Centre, Department of Neurodegenerative Disease, UCL Institute of Neurology, Queen Square, London UK;
• Benedetta Nacmias – Department of Neuroscience, Psychology, Drug Research and Child Health, University of Florence, Florence, Italy;
• Linn Öijerstedt – Center for Alzheimer Research, Division of Neurogeriatrics, Department of Neurobiology, Care Sciences and Society, Bioclinicum, Karolinska Institutet, Solna, Sweden; Unit for Hereditary Dementias, Theme Ageing, Karolinska University Hospital, Solna, Sweden;
• Jaume Olives – Alzheimer's disease and Other Cognitive Disorders Unit, Neurology Service, Hospital Clínic, Barcelona, Spain;
• Sebastien Ourselin – School of Biomedical Engineering & Imaging Sciences, King's College London, London, UK;
• Alessandro Padovani – Centre for Neurodegenerative Disorders, Department of Clinical and Experimental Sciences, University of Brescia, Italy
• Jessica Panman – Department of Neurology, Erasmus Medical Center, Rotterdam, Netherlands;
• Janne M. Papma – Department of Neurology, Erasmus Medical Center, Rotterdam;
• Yolande Pijnenburg – Amsterdam University Medical Centre, Amsterdam VUmc, Amsterdam, Netherlands;
• Cristina Polito – Department of Biomedical, Experimental and Clinical Sciences “Mario Serio”, Nuclear Medicine Unit, University of Florence, Florence, Italy
• Enrico Premi – Stroke Unit, ASST Brescia Hospital, Brescia, Italy
• Sara Prioni – Fondazione IRCCS Istituto Neurologico Carlo Besta, Milano, Italy;
• Catharina Prix ‐Neurologische Klinik, Ludwig‐Maximilians‐Universität München, Munich, Germany;
• Rosa Rademakers [as London Ontario geneticist] – Department of Neurosciences, Mayo Clinic, Jacksonville, Florida, USA;
• Veronica Redaelli ‐Fondazione IRCCS Istituto Neurologico Carlo Besta, Milano, Italy;
• Daisy Rinaldi – Centre de référence des démences rares ou précoces, IM2A, Département de Neurologie, AP‐HP – Hôpital Pitié‐Salpêtrière, Paris, France; Sorbonne Université, Paris Brain Institute – Institut du Cerveau – ICM, Inserm U1127, CNRS UMR 7225, AP‐HP – Hôpital Pitié‐Salpêtrière, Paris, France; Département de Neurologie, AP‐HP – Hôpital Pitié‐Salpêtrière, Paris, France; Reference Network for Rare Neurological Diseases (ERN‐RND);
• Tim Rittman – Department of Clinical Neurosciences, University of Cambridge, Cambridge, UK;
• Ekaterina Rogaeva ‐Tanz Centre for Research in Neurodegenerative Diseases, University of Toronto, Toronto, Canada;
• Adeline Rollin – CHU, CNR‐MAJ, Labex Distalz, LiCEND Lille, France;
• Pedro Rosa‐Neto ‐Translational Neuroimaging Laboratory, McGill Centre for Studies in Ageing, McGill University, Montreal, Québec, Canada;
• Giacomina Rossi – Fondazione IRCCS Istituto Neurologico Carlo Besta, Milano, Italy;
• Martin Rossor – Dementia Research Centre, Department of Neurodegenerative Disease, UCL Institute of Neurology, Queen Square, London, UK;
• Beatriz Santiago – Neurology Department, Centro Hospitalar e Universitario de Coimbra, Coimbra, Portugal;
• Dario Saracino – Sorbonne Université, Paris Brain Institute – Institut du Cerveau – ICM, Inserm U1127, CNRS UMR 7225, AP‐HP – Hôpital Pitié‐Salpêtrière, Paris, France; Inria, Aramis project‐team, F‐75013, Paris, France; Centre de référence des démences rares ou précoces, IM2A, Département de Neurologie, AP‐HP – Hôpital Pitié‐Salpêtrière, Paris, France;
• Sabrina Sayah – Sorbonne Université, Paris Brain Institute – Institut du Cerveau – ICM, Inserm U1127, CNRS UMR 7225, AP‐HP – Hôpital Pitié‐Salpêtrière, Paris, France;
• Elio Scarpini – Fondazione IRCCS Ca′ Granda Ospedale Maggiore Policlinico, Neurodegenerative Diseases Unit, Milan, Italy; University of Milan, Centro Dino Ferrari, Milan, Italy;
• Sonja Schönecker – Neurologische Klinik, Ludwig‐Maximilians‐Universität München, Munich, Germany;
• Harro Seelaar – Department of Neurology, Erasmus Medical Centre, Rotterdam, Netherlands;
• Elisa Semler ‐Department of Neurology, University of Ulm, Ulm;
• Rachelle Shafei – Dementia Research Centre, Department of Neurodegenerative Disease, UCL Institute of Neurology, Queen Square, London, UK;
• Christen Shoesmith – Department of Clinical Neurological Sciences, University of Western Ontario, London, Ontario, Canada;
• Imogen Swift – Department of Neurodegenerative Disease, Dementia Research Centre, UCL Institute of Neurology, Queen Square, London, UK;
• Miguel Tábuas‐Pereira – Neurology Department, Centro Hospitalar e Universitario de Coimbra, Coimbra, Portugal;
• Mikel Tainta – Neuroscience Area, Biodonostia Health Research Insitute, San Sebastian, Gipuzkoa, Spain;
• Ricardo Taipa – Neuropathology Unit and Department of Neurology, Centro Hospitalar do Porto – Hospital de Santo António, Oporto, Portugal;
• David Tang‐Wai ‐The University Health Network, Krembil Research Institute, Toronto, Canada;
• David L Thomas – Neuroimaging Analysis Centre, Department of Brain Repair and Rehabilitation, UCL Institute of Neurology, Queen Square, London, UK;
• Paul Thompson – Division of Neuroscience and Experimental Psychology, Wolfson Molecular Imaging Centre, University of Manchester, Manchester, UK;
• Hakan Thonberg – Center for Alzheimer Research, Division of Neurogeriatrics, Karolinska Institutet, Stockholm, Sweden;
• Carolyn Timberlake – Department of Clinical Neurosciences, University of Cambridge, Cambridge, UK;
• Pietro Tiraboschi – Fondazione IRCCS Istituto Neurologico Carlo Besta, Milano, Italy;
• Emily Todd – Dementia Research Centre, Department of Neurodegenerative Disease, UCL Institute of Neurology, UK;
• Philip Van Damme – Neurology Service, University Hospitals Leuven, Belgium; Laboratory for Neurobiology, VIB‐KU Leuven Centre for Brain Research, Leuven, Belgium;
• Mathieu Vandenbulcke – Geriatric Psychiatry Service, University Hospitals Leuven, Belgium; Neuropsychiatry, Department of Neurosciences, KU Leuven, Leuven, Belgium;
• Michele Veldsman – Nuffield Department of Clinical Neurosciences, Medical Sciences Division, University of Oxford, Oxford, UK;
• Ana Verdelho – Department of Neurosciences and Mental Health, Centro Hospitalar Lisboa Norte – Hospital de Santa Maria & Faculty of Medicine, University of Lisbon, Lisbon, Portugal;
• Jorge Villanua – OSATEK, University of Donostia, San Sebastian, Gipuzkoa, Spain;
• Jason Warren – Dementia Research Centre, Department of Neurodegenerative Disease, UCL Institute of Neurology, Queen Square, London, UK;
• Carlo Wilke – Department of Neurodegenerative Diseases, Hertie‐Institute for Clinical Brain Research and Center of Neurology, University of Tübingen, Tübingen, Germany; Center for Neurodegenerative Diseases (DZNE), Tübingen, Germany;
• Elisabeth Wlasich – Neurologische Klinik, Ludwig‐Maximilians‐Universität München, Munich, Germany;
• Henrik Zetterberg – Dementia Research Institute, Department of Neurodegenerative Disease, UCL Institute of Neurology, Queen Square, London, UK;
• Miren Zulaica – Neuroscience Area, Biodonostia Health Research Insitute, San Sebastian, Gipuzkoa, Spain.

## Supporting information


**Table S1.** Adjusted mean differences with 95% bootstrapped bias‐corrected confidence intervals in the comparison of CBI‐R total scores between healthy controls and each of the genetic groups stratified by global CDR plus NACC FTLD score.
**Table S2.** Adjusted mean differences in each of the ten CBI‐R domains scores between the genetic groups stratified by global CDR plus NACC FTLD scores, with 95% bootstrapped bias‐corrected confidence intervals.
**Table S3.** Adjusted mean differences from within‐group analysis of each of the ten CBI‐R domains for symptomatic mutation carriers ((A) *C9orf72*, (B) *GRN*, (C) *MAPT*) with 95% bootstrapped bias‐corrected confidence intervals.
**Table S4.** Partial correlations between scores in the 10 domains of the CBI‐R and volumes of neuroanatomical regions of interest adjusting for disease severity and age (*r* values and corresponding *p* values are shown) for the *C9orf72* mutation carriers.
**Table S5.** Partial correlations between scores in the 10 domains of the CBI‐R and volumes of neuroanatomical regions of interest adjusting for disease severity and age (*r* values and corresponding *p* values are shown) for the *GRN* mutation carriers.
**Table S6.** Partial correlations between scores in the 10 domains of the CBI‐R and volumes of neuroanatomical regions of interest adjusting for disease severity and age (*r* values and corresponding *p* values are shown) for the *MAPT* mutation carriers.
**Figure S1.** Correlations between CBI‐R total scores and (i) on the left, CDR plus NACC FTLD sum of boxes scores [*C9orf72* (*r* = 0.78, *p* < 0.001), *GRN* (*r* = 0.82, *p* < 0.001) and *MAPT* (*r* = 0.60, *p* < 0.001)], and (ii) on the right, FRS scores [C9orf72 (*r* = −0.92, *p* < 0.001), GRN (*r* = −0.88, *p* < 0.001) and MAPT (*r* = −0.88, *p* < 0.001)].
**Figure S2.** CBI‐R individual domain scores (as a percentage) in each of the ten domains in all symptomatic mutation carrier groups: (A) *C9orf72*, (B) *GRN*, (C) *MAPT*. The error bars represent standard error of the mean.
