# Supplementary material for: The CBI‐R detects early behavioural impairment in genetic frontotemporal dementia
Source: Ann Clin Transl Neurol. 2022 Mar 26;9(5):644–58. doi: 10.1002/acn3.51544 (PMC9082390; doi:10.1002/acn3.51544)
Supplement: Supplementary file 1 — Table S1. Adjusted mean differences with 95% bootstrapped bias‐corrected confidence intervals in the comparison of CBI‐R total scores between healthy controls and each of the genetic groups stratified by global CDR plus NACC FTLD score. Table S2. Adjusted mean differences in each of the ten CBI‐R domains scores between the genetic groups stratified by global CDR plus NACC FTLD scores, with 95% bootstrapped bias‐corrected confidence intervals. Table S3. Adjusted mean differences from within‐group analysis of each of the ten CBI‐R domains for symptomatic mutation carriers ((A) C9orf72, (B) GRN, (C) MAPT) with 95% bootstrapped bias‐corrected confidence intervals. Table S4. Partial correlations between scores in the 10 domains of the CBI‐R and volumes of neuroanatomical regions of interest adjusting for disease severity and age (r values and corresponding p values are shown) for the C9orf72 mutation carriers. Table S5. Partial correlations between scores in the 10 domains of the CBI‐R and volumes of neuroanatomical regions of interest adjusting for disease severity and age (r values and corresponding p values are shown) for the GRN mutation carriers. Table S6. Partial correlations between scores in the 10 domains of the CBI‐R and volumes of neuroanatomical regions of interest adjusting for disease severity and age (r values and corresponding p values are shown) for the MAPT mutation carriers. Figure S1. Correlations between CBI‐R total scores and (i) on the left, CDR plus NACC FTLD sum of boxes scores [C9orf72 (r = 0.78, p < 0.001), GRN (r = 0.82, p < 0.001) and MAPT (r = 0.60, p < 0.001)], and (ii) on the right, FRS scores [C9orf72 (r = −0.92, p < 0.001), GRN (r = −0.88, p < 0.001) and MAPT (r = −0.88, p < 0.001)]. Figure S2. CBI‐R individual domain scores (as a percentage) in each of the ten domains in all symptomatic mutation carrier groups: (A) C9orf72, (B) GRN, (C) MAPT. The error bars represent standard error of the mean. [file ACN3-9-644-s001.docx]

**Supplementary Data**

**Table S1: Adjusted mean differences with 95% bootstrapped bias-corrected confidence intervals in the comparison of CBI-R total scores between healthy controls and each of the genetic groups stratified by global CDR plus NACC FTLD score. Significant values are shown in bold.**

| **CBI-R Total** | | **Healthy controls** | **C9orf72** | | | | | | **GRN** | | | | | | **MAPT** | | | | | |
| --- | --- | --- | --- | --- | --- | --- | --- | --- | --- | --- | --- | --- | --- | --- | --- | --- | --- | --- | --- | --- |
|  |  |  | 0 | | 0.5 | | **1+** | | 0 | | 0.5 | | **1+** | | 0 | | 0.5 | | **1+** | |
| **Healthy controls** | |  | 0.62 | | **7.27** | | **62.05** | | -1.13 | | **6.98** | | **47.59** | | **4.63** | | 3.90 | | **54.69** | |
|  |  |  | -1.39 | 2.62 | **2.35** | **12.18** | **55.09** | **69.02** | -2.51 | 0.26 | **2.50** | **11.47** | **37.65** | **57.53** | **0.49** | **8.77** | -1.90 | 9.69 | **40.13** | **69.26** |
| **C9orf72** | **0** |  |  |  | **6.65** | | **61.44** | | -1.74 | | **6.37** | | **46.98** | | 4.02 | | 3.28 | | **54.08** | |
|  |  |  |  |  | **1.56** | **11.74** | **54.54** | **68.34** | -3.89 | 0.40 | **1.61** | **11.12** | **37.09** | **56.86** | -0.37 | 8.41 | -2.65 | 9.21 | **39.41** | **68.74** |
|  | **0.5** |  |  | |  |  | **54.79** | | **-8.40** | | -0.29 | | **40.32** | | -2.64 | | -3.37 | | **47.43** | |
|  |  |  |  |  |  |  | **46.62** | **62.95** | **-13.40** | **-3.40** | -6.76 | 6.19 | **29.67** | **50.98** | -8.93 | 3.66 | -10.83 | 4.09 | **32.10** | **62.75** |
|  | **1+** |  |  | |  | |  |  | **-63.18** | | **-55.07** | | **-14.46** | | **-57.42** | | **-58.16** | | -7.36 | |
|  |  |  |  |  |  |  |  |  | **-70.17** | **-56.20** | **-63.10** | **-47.04** | **-26.07** | **-2.85** | **-65.72** | **-49.12** | **-66.98** | **-49.33** | -22.84 | 8.12 |
| **GRN** | **0** |  |  | |  | |  | |  |  | **8.11** | | **48.72** | | **5.76** | | 5.02 | | **55.82** | |
|  |  |  |  |  |  |  |  |  |  |  | **3.50** | **12.72** | **38.88** | **58.56** | **1.52** | **10.00** | -0.83 | 10.88 | **41.23** | **70.42** |
|  | **0.5** |  |  | |  | |  | |  | |  |  | **40.61** | | -2.35 | | 0.00 | | **47.71** | |
|  |  |  |  |  |  |  |  |  |  |  |  |  | **29.76** | **51.46** | -8.42 | 3.72 | -10.31 | 4.14 | **32.74** | **62.68** |
|  | **1+** |  |  | |  | |  | |  | |  | |  |  | **-42.96** | | **-43.70** | | 7.10 | |
|  |  |  |  |  |  |  |  |  |  |  |  |  |  |  | **-53.54** | **-32.38** | **-54.84** | **-32.55** | -10.06 | 24.27 |
| **MAPT** | **0** |  |  | |  | |  | |  | |  | |  | |  |  | -0.74 | | **50.06** | |
|  |  |  |  |  |  |  |  |  |  |  |  |  |  |  |  |  | -7.20 | 5.73 | **34.77** | **65.35** |
|  | **0.5** |  |  | |  | |  | |  | |  | |  | |  | |  |  | **50.80** | |
|  |  |  |  |  |  |  |  |  |  |  |  |  |  |  |  |  |  |  | **36.08** | **65.52** |
|  | **1+** |  |  | |  | |  | |  | |  | |  | |  | |  | |  |  |
|  |  |  |  |  |  |  |  |  |  |  |  |  |  |  |  |  |  |  |  |  |

**Table S2 a-j: Adjusted mean differences in each of the ten CBI-R domains scores between the genetic groups stratified by global CDR plus NACC FTLD scores, with 95% bootstrapped bias-corrected confidence intervals. Significant values are shown in bold. a) Motivation, b) Stereotypic and Motor Behaviours, c) Eating Habits, d) Abnormal Behaviour, e) Beliefs, f) Mood, g) Sleep, h) Everyday Skills, i) Self Care, j) Memory and Orientation.**

**a)**

| **Motivation** | | **Healthy controls** | **C9orf72** | | | | | | **GRN** | | | | | | **MAPT** | | | | | |
| --- | --- | --- | --- | --- | --- | --- | --- | --- | --- | --- | --- | --- | --- | --- | --- | --- | --- | --- | --- | --- |
|  |  |  | **0** | | **0.5** | | **1+** | | **0** | | **0.5** | | **1+** | | **0** | | **0.5** | | **1+** | |
| **Healthy controls** | |  | 0.14 | | 1.05 | | **9.19** | | -0.15 | | **1.55** | | **8.70** | | 0.90 | | 0.58 | | **8.64** | |
|  |  |  | -0.43 | 0.71 | -0.29 | 2.39 | **7.67** | **10.72** | -0.43 | 0.12 | **0.14** | **2.97** | **6.85** | **10.55** | -0.03 | 1.83 | -0.76 | 1.91 | **5.45** | **11.83** |
| **C9orf72** | **0** |  |  |  | 0.91 | | **9.05** | | -0.29 | | 1.41 | | **8.56** | | 0.76 | | 0.43 | | **8.50** | |
|  |  |  |  |  | -0.54 | 2.36 | **7.49** | **10.61** | -0.84 | 0.25 | -0.08 | 2.91 | **6.74** | **10.38** | -0.22 | 1.74 | -0.95 | 1.82 | **5.24** | **11.77** |
|  | **0.5** |  |  | |  |  | **8.14** | | -1.20 | | 0.51 | | **7.65** | | -0.15 | | -0.47 | | **7.59** | |
|  |  |  |  |  |  |  | **6.20** | **10.09** | -2.55 | 0.15 | -1.40 | 2.41 | **5.46** | **9.84** | -1.73 | 1.44 | -2.34 | 1.39 | **4.06** | **11.13** |
|  | **1+** |  |  | |  | |  |  | **-9.34** | | **-7.64** | | -0.49 | | **-8.29** | | **-8.62** | | -0.55 | |
|  |  |  |  |  |  |  |  |  | **-10.86** | **-7.83** | **-9.66** | **-5.62** | -2.74 | 1.76 | **-10.04** | **-6.54** | **-10.62** | **-6.61** | -4.02 | 2.92 |
| **GRN** | **0** |  |  | |  | |  | |  |  | **1.71** | | **8.85** | | **1.05** | | 0.73 | | **8.79** | |
|  |  |  |  |  |  |  |  |  |  |  | **0.28** | **3.13** | **7.05** | **10.65** | **0.13** | **1.98** | -0.62 | 2.07 | **5.60** | **11.99** |
|  | **0.5** |  |  | |  | |  | |  | |  |  | **7.15** | | -0.65 | | 0.00 | | **7.09** | |
|  |  |  |  |  |  |  |  |  |  |  |  |  | **4.91** | **9.39** | -2.31 | 1.00 | -2.89 | 0.93 | **3.70** | **10.48** |
|  | **1+** |  |  | |  | |  | |  | |  | |  |  | **-7.80** | | **-8.13** | | -0.06 | |
|  |  |  |  |  |  |  |  |  |  |  |  |  |  |  | **-9.76** | **-5.84** | **-10.36** | **-5.9** | -3.74 | 3.62 |
| **MAPT** | **0** |  |  | |  | |  | |  | |  | |  | |  |  | -0.33 | | **7.74** | |
|  |  |  |  |  |  |  |  |  |  |  |  |  |  |  |  |  | -1.76 | 1.11 | **4.33** | **11.16** |
|  | **0.5** |  |  | |  | |  | |  | |  | |  | |  | |  |  | **8.07** | |
|  |  |  |  |  |  |  |  |  |  |  |  |  |  |  |  |  |  |  | **4.73** | **11.40** |
|  | **1+** |  |  | |  | |  | |  | |  | |  | |  | |  | |  |  |
|  |  |  |  |  |  |  |  |  |  |  |  |  |  |  |  |  |  |  |  |  |

**b)**

| **Stereotypic Behaviour** | | **Healthy controls** | **C9orf72** | | | | | | | | | | | **GRN** | | | | | | | | | | | | **MAPT** | | | | | | | | | | |
| --- | --- | --- | --- | --- | --- | --- | --- | --- | --- | --- | --- | --- | --- | --- | --- | --- | --- | --- | --- | --- | --- | --- | --- | --- | --- | --- | --- | --- | --- | --- | --- | --- | --- | --- | --- | --- |
|  |  |  | **0** | | | **0.5** | | | | **1+** | | | | **0** | | | | **0.5** | | | | **1+** | | | | **0** | | | | **0.5** | | | | **1+** | | |
| **Healthy controls** | |  | 0.14 | | | **0.89** | | | | **5.71** | | | | -0.06 | | | | **0.78** | | | | **2.88** | | | | **0.92** | | | | 0.48 | | | | **6.85** | | |
|  |  |  | -0.19 | 0.46 | **0.16** | | **1.61** | | **4.57** | | **6.85** | | -0.32 | | 0.21 | | **0.02** | | **1.54** | | **1.85** | | **3.91** | | **0.14** | | **1.69** | | -0.16 | | 1.12 | | **4.99** | | **8.71** | |
| **C9orf72** | **0** |  |  |  | **0.75** | | | | **5.57** | | | | -0.19 | | | | 0.64 | | | | **2.74** | | | | 0.78 | | | | 0.34 | | | | **6.71** | | | |
|  |  |  |  |  | **0.00** | | **1.50** | | **4.35** | | **6.78** | | -0.59 | | 0.20 | | -0.19 | | 1.48 | | **1.66** | | **3.82** | | -0.04 | | 1.60 | | -0.35 | | 1.03 | | **4.83** | | **8.60** | |
|  | **0.5** |  |  | | |  | |  | | **4.82** | | | | **-0.94** | | | | -0.11 | | | | **1.99** | | | | 0.03 | | | | -0.41 | | | | **5.96** | | |
|  |  |  |  |  |  |  | |  | | **3.46** | | **6.18** | | **-1.71** | | **-0.18** | | -1.13 | | 0.92 | | **0.77** | | **3.21** | | -1.02 | | 1.08 | | -1.34 | | 0.52 | | **3.98** | | **7.95** |
|  | **1+** |  |  | | |  | | | |  | |  | | **-5.76** | | | | **-4.93** | | | | **-2.83** | | | | **-4.79** | | | | **-5.23** | | | | 1.15 | | |
|  |  |  |  |  |  |  |  |  |  |  | |  | | **-6.93** | | **-4.60** | | **-6.30** | | **-3.55** | | **-4.34** | | **-1.32** | | **-6.18** | | **-3.40** | | **-6.51** | | **-3.95** | | -0.99 | | 3.28 |
| **GRN** | **0** |  |  | | |  | | | |  | | | |  | |  | | **0.84** | | | | **2.93** | | | | **0.97** | | | | 0.54 | | | | **6.91** | | |
|  |  |  |  |  |  |  |  |  |  |  |  |  |  |  | |  | | **0.03** | | **1.65** | | **1.87** | | **4.00** | | **0.18** | | **1.77** | | -0.14 | | 1.21 | | **5.04** | | **8.78** |
|  | **0.5** |  |  | | |  | | | |  | | | |  | | | |  | |  | | **2.10** | | | | 0.14 | | | | 0.00 | | | | **6.07** | | |
|  |  |  |  |  |  |  |  |  |  |  |  |  |  |  |  |  |  |  | |  | | **0.78** | | **3.42** | | -0.95 | | 1.22 | | -1.28 | | 0.68 | | **4.07** | | **8.07** |
|  | **1+** |  |  | | |  | | | |  | | | |  | | | |  | | | |  | |  | | **-1.96** | | | | **-2.40** | | | | **3.97** | | |
|  |  |  |  |  |  |  |  |  |  |  |  |  |  |  |  |  |  |  |  |  |  |  | |  | | **-3.26** | | **-0.66** | | **-3.57** | | **-1.23** | | **1.89** | | **6.06** |
| **MAPT** | **0** |  |  | | |  | | | |  | | | |  | | | |  | | | |  | | | |  | |  | | -0.44 | | | | **5.94** | | |
|  |  |  |  |  |  |  |  |  |  |  |  |  |  |  |  |  |  |  |  |  |  |  |  |  |  |  | |  | | -1.31 | | 0.44 | | **3.85** | | **8.02** |
|  | **0.5** |  |  | | |  | | | |  | | | |  | | | |  | | | |  | | | |  | | | |  | |  | | **6.37** | | |
|  |  |  |  |  |  |  |  |  |  |  |  |  |  |  |  |  |  |  |  |  |  |  |  |  |  |  |  |  |  |  | |  | | **4.53** | | **8.22** |
|  | **1+** |  |  | | |  | | | |  | | | |  | | | |  | | | |  | | | |  | | | |  | | | |  | |  |
|  |  |  |  |  |  |  |  |  |  |  |  |  |  |  |  |  |  |  |  |  |  |  |  |  |  |  |  |  |  |  |  |  |  |  | |  |

**c)**

| **Eating Habits** | | **Healthy controls** | **C9orf72** | | | | | | **GRN** | | | | | | **MAPT** | | | | | |
| --- | --- | --- | --- | --- | --- | --- | --- | --- | --- | --- | --- | --- | --- | --- | --- | --- | --- | --- | --- | --- |
|  |  |  | **0** | | **0.5** | | **1+** | | **0** | | **0.5** | | **1+** | | **0** | | **0.5** | | **1+** | |
| **Healthy controls** | |  | -0.06 | | 0.51 | | **5.94** | | -0.07 | | 0.50 | | **4.53** | | 0.28 | | 0.18 | | **6.07** | |
|  |  |  | -0.34 | 0.22 | -0.33 | 1.35 | **4.74** | **7.14** | -0.24 | 0.10 | -0.45 | 1.45 | **3.03** | **6.03** | -0.26 | 0.83 | -0.52 | 0.89 | **3.59** | **8.54** |
| **C9orf72** | **0** |  |  |  | 0.57 | | **6.00** | | -0.01 | | 0.56 | | **4.58** | | 0.34 | | 0.24 | | **6.12** | |
|  |  |  |  |  | -0.31 | 1.44 | **4.76** | **7.24** | -0.32 | 0.29 | -0.36 | 1.47 | **3.09** | **6.07** | -0.26 | 0.94 | -0.50 | 0.98 | **3.65** | **8.60** |
|  | **0.5** |  |  | |  |  | **5.43** | | -0.58 | | -0.01 | | **4.02** | | -0.23 | | -0.33 | | **5.56** | |
|  |  |  |  |  |  |  | **3.98** | **6.89** | -1.44 | 0.28 | -1.20 | 1.18 | **2.35** | **5.68** | -1.23 | 0.78 | -1.43 | 0.77 | **2.92** | **8.19** |
|  | **1+** |  |  | |  | |  |  | **-6.01** | | **-5.44** | | -1.42 | | **-5.66** | | **-5.76** | | 0.12 | |
|  |  |  |  |  |  |  |  |  | **-7.21** | **-4.81** | **-6.98** | **-3.91** | -3.28 | 0.45 | **-7.00** | **-4.32** | **-7.14** | **-4.38** | -2.62 | 2.86 |
| **GRN** | **0** |  |  | |  | |  | |  |  | 0.57 | | **4.60** | | 0.35 | | 0.25 | | **6.14** | |
|  |  |  |  |  |  |  |  |  |  |  | -0.39 | 1.52 | **3.11** | **6.08** | -0.19 | 0.89 | -0.45 | 0.95 | **3.67** | **8.60** |
|  | **0.5** |  |  | |  | |  | |  | |  |  | **4.03** | | -0.22 | | 0.00 | | **5.57** | |
|  |  |  |  |  |  |  |  |  |  |  |  |  | **2.21** | **5.85** | -1.31 | 0.88 | -1.53 | 0.90 | **2.93** | **8.20** |
|  | **1+** |  |  | |  | |  | |  | |  | |  |  | **-4.24** | | **-4.34** | | 1.54 | |
|  |  |  |  |  |  |  |  |  |  |  |  |  |  |  | **-5.84** | **-2.65** | **-5.95** | **-2.74** | -1.30 | 4.38 |
| **MAPT** | **0** |  |  | |  | |  | |  | |  | |  | |  |  | -0.10 | | **5.78** | |
|  |  |  |  |  |  |  |  |  |  |  |  |  |  |  |  |  | -0.95 | 0.74 | **3.27** | **8.30** |
|  | **0.5** |  |  | |  | |  | |  | |  | |  | |  | |  |  | **5.88** | |
|  |  |  |  |  |  |  |  |  |  |  |  |  |  |  |  |  |  |  | **3.38** | **8.39** |
|  | **1+** |  |  | |  | |  | |  | |  | |  | |  | |  | |  |  |
|  |  |  |  |  |  |  |  |  |  |  |  |  |  |  |  |  |  |  |  |  |

**d)**

| **Abnormal Behaviour** | | **Healthy controls** | **C9orf72** | | | | | | **GRN** | | | | | | **MAPT** | | | | | |
| --- | --- | --- | --- | --- | --- | --- | --- | --- | --- | --- | --- | --- | --- | --- | --- | --- | --- | --- | --- | --- |
|  |  |  | **0** | | **0.5** | | **1+** | | **0** | | **0.5** | | **1+** | | **0** | | **0.5** | | **1+** | |
| **Healthy controls** | |  | 0.29 | | **1.10** | | **7.09** | | -0.13 | | 0.68 | | **5.05** | | 0.40 | | 0.62 | | **6.45** | |
|  |  |  | -0.10 | 0.69 | **0.13** | **2.07** | **5.73** | **8.44** | -0.40 | 0.15 | -0.23 | 1.60 | **3.58** | **6.52** | -0.29 | 1.09 | -0.27 | 1.52 | **4.36** | **8.54** |
| **C9orf72** | **0** |  |  |  | 0.80 | | **6.79** | | **-0.42** | | 0.39 | | **4.76** | | 0.11 | | 0.33 | | **6.16** | |
|  |  |  |  |  | -0.23 | 1.84 | **5.47** | **8.12** | **-0.82** | **-0.02** | -0.57 | 1.35 | **3.28** | **6.23** | -0.67 | 0.89 | -0.61 | 1.27 | **4.09** | **8.23** |
|  | **0.5** |  |  | |  |  | **5.99** | | **-1.22** | | -0.41 | | **3.95** | | -0.69 | | -0.47 | | **5.35** | |
|  |  |  |  |  |  |  | **4.36** | **7.61** | **-2.20** | **-0.25** | -1.72 | 0.89 | **2.29** | **5.61** | -1.89 | 0.50 | -1.77 | 0.82 | **3.06** | **7.65** |
|  | **1+** |  |  | |  | |  |  | **-7.21** | | **-6.40** | | **-2.04** | | **-6.68** | | **-6.46** | | -0.63 | |
|  |  |  |  |  |  |  |  |  | **-8.55** | **-5.87** | **-8.04** | **-4.76** | **-3.97** | **-0.10** | **-8.20** | **-5.16** | **-8.02** | **-4.91** | -3.06 | 1.80 |
| **GRN** | **0** |  |  | |  | |  | |  |  | 0.81 | | **5.17** | | 0.53 | | 0.75 | | **6.58** | |
|  |  |  |  |  |  |  |  |  |  |  | -0.07 | 1.69 | **3.77** | **6.58** | -0.19 | 1.25 | -0.15 | 1.65 | **4.50** | **8.66** |
|  | **0.5** |  |  | |  | |  | |  | |  |  | **4.36** | | -0.28 | | 0.00 | | **5.77** | |
|  |  |  |  |  |  |  |  |  |  |  |  |  | **2.68** | **6.05** | -1.40 | 0.84 | -1.30 | 1.18 | **3.52** | **8.02** |
|  | **1+** |  |  | |  | |  | |  | |  | |  |  | **-4.65** | | **-4.42** | | 1.40 | |
|  |  |  |  |  |  |  |  |  |  |  |  |  |  |  | **-6.26** | **-3.03** | **-6.07** | **-2.78** | -1.03 | 3.84 |
| **MAPT** | **0** |  |  | |  | |  | |  | |  | |  | |  |  | 0.22 | | **6.05** | |
|  |  |  |  |  |  |  |  |  |  |  |  |  |  |  |  |  | -0.65 | 1.09 | **3.85** | **8.25** |
|  | **0.5** |  |  | |  | |  | |  | |  | |  | |  | |  |  | **5.83** | |
|  |  |  |  |  |  |  |  |  |  |  |  |  |  |  |  |  |  |  | **3.60** | **8.06** |
|  | **1+** |  |  | |  | |  | |  | |  | |  | |  | |  | |  |  |
|  |  |  |  |  |  |  |  |  |  |  |  |  |  |  |  |  |  |  |  |  |

**e)**

| **Beliefs** | | **Healthy controls** | **C9orf72** | | | | | | **GRN** | | | | | | **MAPT** | | | | | |
| --- | --- | --- | --- | --- | --- | --- | --- | --- | --- | --- | --- | --- | --- | --- | --- | --- | --- | --- | --- | --- |
|  |  |  | **0** | | **0.5** | | **1+** | | **0** | | **0.5** | | **1+** | | **0** | | **0.5** | | **1+** | |
| **Healthy controls** | |  | -0.02 | | 0.04 | | **1.44** | | 0.04 | | -0.14 | | **0.93** | | 0.00 | | -0.03 | | **0.34** | |
|  |  |  | -0.11 | 0.07 | -0.10 | 0.17 | **0.87** | **2.01** | -0.04 | 0.13 | -0.33 | 0.06 | **0.31** | **1.55** | -0.07 | 0.07 | -0.24 | 0.18 | **0.02** | **0.65** |
| **C9orf72** | **0** |  |  |  | 0.06 | | **1.46** | | 0.07 | | -0.12 | | **0.95** | | 0.02 | | -0.01 | | **0.36** | |
|  |  |  |  |  | -0.09 | 0.21 | **0.89** | **2.03** | -0.05 | 0.19 | -0.32 | 0.09 | **0.34** | **1.57** | -0.08 | 0.12 | -0.22 | 0.21 | **0.03** | **0.68** |
|  | **0.5** |  |  | |  |  | **1.41** | | 0.01 | | -0.17 | | **0.89** | | -0.04 | | -0.07 | | 0.30 | |
|  |  |  |  |  |  |  | **0.82** | **1.99** | -0.16 | 0.18 | -0.40 | 0.05 | **0.26** | **1.52** | -0.19 | 0.11 | -0.30 | 0.17 | -0.04 | 0.63 |
|  | **1+** |  |  | |  | |  |  | **-1.40** | | **-1.58** | | -0.51 | | **-1.44** | | **-1.47** | | **-1.11** | |
|  |  |  |  |  |  |  |  |  | **-1.97** | **-0.82** | **-2.18** | **-0.98** | -1.37 | 0.34 | **-2.01** | **-0.87** | **-2.08** | **-0.9** | **-1.75** | **-0.46** |
| **GRN** | **0** |  |  | |  | |  | |  |  | -0.18 | | **0.88** | | -0.05 | | -0.07 | | 0.29 | |
|  |  |  |  |  |  |  |  |  |  |  | -0.41 | 0.04 | **0.30** | **1.47** | -0.14 | 0.05 | -0.31 | 0.16 | -0.06 | 0.64 |
|  | **0.5** |  |  | |  | |  | |  | |  |  | **1.07** | | 0.14 | | 0.00 | | **0.47** | |
|  |  |  |  |  |  |  |  |  |  |  |  |  | **0.42** | **1.72** | -0.07 | 0.34 | -0.14 | 0.36 | **0.14** | **0.81** |
|  | **1+** |  |  | |  | |  | |  | |  | |  |  | **-0.93** | | **-0.96** | | -0.59 | |
|  |  |  |  |  |  |  |  |  |  |  |  |  |  |  | **-1.54** | **-0.32** | **-1.59** | **-0.3** | -1.29 | 0.10 |
| **MAPT** | **0** |  |  | |  | |  | |  | |  | |  | |  |  | -0.03 | | 0.34 | |
|  |  |  |  |  |  |  |  |  |  |  |  |  |  |  |  |  | -0.24 | 0.18 | -0.01 | 0.68 |
|  | **0.5** |  |  | |  | |  | |  | |  | |  | |  | |  |  | **0.37** | |
|  |  |  |  |  |  |  |  |  |  |  |  |  |  |  |  |  |  |  | **0.04** | **0.70** |
|  | **1+** |  |  | |  | |  | |  | |  | |  | |  | |  | |  |  |
|  |  |  |  |  |  |  |  |  |  |  |  |  |  |  |  |  |  |  |  |  |

**f)**

| **Mood** | | **Healthy controls** | **C9orf72** | | | | | | **GRN** | | | | | | **MAPT** | | | | | |
| --- | --- | --- | --- | --- | --- | --- | --- | --- | --- | --- | --- | --- | --- | --- | --- | --- | --- | --- | --- | --- |
|  |  |  | **0** | | **0.5** | | **1+** | | **0** | | **0.5** | | **1+** | | **0** | | **0.5** | | **1+** | |
| **Healthy controls** | |  | -0.06 | | 0.69 | | **3.27** | | **-0.33** | | 0.69 | | **2.97** | | **0.86** | | 0.42 | | **2.89** | |
|  |  |  | -0.45 | 0.32 | -0.07 | 1.45 | **2.47** | **4.07** | **-0.66** | **-0.01** | -0.02 | 1.40 | **2.02** | **3.91** | **0.07** | **1.65** | -0.51 | 1.35 | **1.72** | **4.05** |
| **C9orf72** | **0** |  |  |  | 0.76 | | **3.34** | | -0.27 | | **0.75** | | **3.03** | | **0.92** | | 0.49 | | **2.95** | |
|  |  |  |  |  | -0.02 | 1.53 | **2.54** | **4.13** | -0.66 | 0.12 | **0.01** | **1.50** | **2.09** | **3.97** | **0.09** | **1.75** | -0.47 | 1.45 | **1.79** | **4.12** |
|  | **0.5** |  |  | |  |  | **2.58** | | **-1.03** | | -0.01 | | **2.27** | | 0.16 | | -0.27 | | **2.19** | |
|  |  |  |  |  |  |  | **1.56** | **3.60** | **-1.79** | **-0.27** | -0.99 | 0.98 | **1.15** | **3.40** | -0.92 | 1.25 | -1.44 | 0.89 | **0.87** | **3.52** |
|  | **1+** |  |  | |  | |  |  | **-3.60** | | **-2.58** | | -0.30 | | **-2.41** | | **-2.85** | | -0.38 | |
|  |  |  |  |  |  |  |  |  | **-4.41** | **-2.80** | **-3.57** | **-1.60** | -1.44 | 0.83 | **-3.54** | **-1.29** | **-4.06** | **-1.6** | -1.68 | 0.92 |
| **GRN** | **0** |  |  | |  | |  | |  |  | **1.02** | | **3.30** | | **1.19** | | 0.75 | | **3.22** | |
|  |  |  |  |  |  |  |  |  |  |  | **0.31** | **1.73** | **2.38** | **4.23** | **0.38** | **2.01** | -0.19 | 1.70 | **2.06** | **4.39** |
|  | **0.5** |  |  | |  | |  | |  | |  |  | **2.28** | | 0.17 | | 0.00 | | **2.20** | |
|  |  |  |  |  |  |  |  |  |  |  |  |  | **1.22** | **3.34** | -0.88 | 1.23 | -1.39 | 0.85 | **0.91** | **3.49** |
|  | **1+** |  |  | |  | |  | |  | |  | |  |  | **-2.11** | | **-2.55** | | -0.08 | |
|  |  |  |  |  |  |  |  |  |  |  |  |  |  |  | **-3.33** | **-0.88** | **-3.81** | **-1.3** | -1.53 | 1.37 |
| **MAPT** | **0** |  |  | |  | |  | |  | |  | |  | |  |  | -0.44 | | **2.03** | |
|  |  |  |  |  |  |  |  |  |  |  |  |  |  |  |  |  | -1.59 | 0.72 | **0.54** | **3.52** |
|  | **0.5** |  |  | |  | |  | |  | |  | |  | |  | |  |  | **2.47** | |
|  |  |  |  |  |  |  |  |  |  |  |  |  |  |  |  |  |  |  | **0.97** | **3.96** |
|  | **1+** |  |  | |  | |  | |  | |  | |  | |  | |  | |  |  |
|  |  |  |  |  |  |  |  |  |  |  |  |  |  |  |  |  |  |  |  |  |

**g)**

| **Sleep** | | **Healthy controls** | **C9orf72** | | | | | | **GRN** | | | | | | **MAPT** | | | | | |
| --- | --- | --- | --- | --- | --- | --- | --- | --- | --- | --- | --- | --- | --- | --- | --- | --- | --- | --- | --- | --- |
|  |  |  | **0** | | **0.5** | | **1+** | | **0** | | **0.5** | | **1+** | | **0** | | **0.5** | | **1+** | |
| **Healthy controls** | |  | -0.11 | | 0.45 | | **2.11** | | -0.19 | | 0.33 | | **2.02** | | **0.49** | | -0.10 | | **0.92** | |
|  |  |  | -0.46 | 0.25 | -0.17 | 1.08 | **1.54** | **2.68** | -0.42 | 0.04 | -0.17 | 0.84 | **1.25** | **2.80** | **0.01** | **0.97** | -0.56 | 0.35 | **0.24** | **1.60** |
| **C9orf72** | **0** |  |  |  | 0.56 | | **2.22** | | -0.08 | | 0.44 | | **2.13** | | **0.60** | | 0.00 | | **1.03** | |
|  |  |  |  |  | -0.10 | 1.21 | **1.60** | **2.84** | -0.43 | 0.27 | -0.13 | 1.01 | **1.32** | **2.93** | **0.06** | **1.13** | -0.51 | 0.52 | **0.29** | **1.76** |
|  | **0.5** |  |  | |  |  | **1.66** | | **-0.64** | | -0.12 | | **1.57** | | 0.04 | | -0.55 | | 0.47 | |
|  |  |  |  |  |  |  | **0.83** | **2.49** | **-1.26** | **-0.02** | -0.89 | 0.66 | **0.62** | **2.52** | -0.70 | 0.78 | -1.29 | 0.18 | -0.43 | 1.37 |
|  | **1+** |  |  | |  | |  |  | **-2.30** | | **-1.78** | | -0.09 | | **-1.62** | | **-2.21** | | **-1.19** | |
|  |  |  |  |  |  |  |  |  | **-2.87** | **-1.73** | **-2.47** | **-1.08** | -0.96 | 0.78 | **-2.34** | **-0.90** | **-2.91** | **-1.52** | **-2.01** | **-0.36** |
| **GRN** | **0** |  |  | |  | |  | |  |  | **0.52** | | **2.21** | | **0.68** | | 0.08 | | **1.11** | |
|  |  |  |  |  |  |  |  |  |  |  | **0.06** | **0.98** | **1.41** | **3.01** | **0.18** | **1.18** | -0.38 | 0.55 | **0.43** | **1.79** |
|  | **0.5** |  |  | |  | |  | |  | |  |  | **1.69** | | 0.16 | | 0.00 | | 0.59 | |
|  |  |  |  |  |  |  |  |  |  |  |  |  | **0.74** | **2.64** | -0.54 | 0.85 | -1.08 | 0.20 | -0.20 | 1.38 |
|  | **1+** |  |  | |  | |  | |  | |  | |  |  | **-1.53** | | **-2.13** | | **-1.10** | |
|  |  |  |  |  |  |  |  |  |  |  |  |  |  |  | **-2.38** | **-0.68** | **-2.98** | **-1.27** | **-2.08** | **-0.12** |
| **MAPT** | **0** |  |  | |  | |  | |  | |  | |  | |  |  | -0.59 | | 0.43 | |
|  |  |  |  |  |  |  |  |  |  |  |  |  |  |  |  |  | -1.20 | 0.01 | -0.35 | 1.21 |
|  | **0.5** |  |  | |  | |  | |  | |  | |  | |  | |  |  | **1.03** | |
|  |  |  |  |  |  |  |  |  |  |  |  |  |  |  |  |  |  |  | **0.23** | **1.82** |
|  | **1+** |  |  | |  | |  | |  | |  | |  | |  | |  | |  |  |
|  |  |  |  |  |  |  |  |  |  |  |  |  |  |  |  |  |  |  |  |  |

**h)**

| **Everyday Skills** | | **Healthy controls** | **C9orf72** | | | | | | **GRN** | | | | | | **MAPT** | | | | | |
| --- | --- | --- | --- | --- | --- | --- | --- | --- | --- | --- | --- | --- | --- | --- | --- | --- | --- | --- | --- | --- |
|  |  |  | **0** | | **0.5** | | **1+** | | **0** | | **0.5** | | **1+** | | **0** | | **0.5** | | **1+** | |
| **Healthy controls** | |  | -0.06 | | 0.21 | | **8.32** | | -0.05 | | 0.13 | | **6.54** | | 0.11 | | -0.07 | | **6.29** | |
|  |  |  | -0.32 | 0.19 | -0.37 | 0.80 | **6.87** | **9.77** | -0.28 | 0.18 | -0.43 | 0.68 | **4.57** | **8.50** | -0.29 | 0.51 | -0.47 | 0.32 | **3.41** | **9.17** |
| **C9orf72** | **0** |  |  |  | 0.28 | | **8.38** | | 0.02 | | 0.19 | | **6.60** | | 0.17 | | -0.01 | | **6.35** | |
|  |  |  |  |  | -0.31 | 0.86 | **6.91** | **9.85** | -0.28 | 0.32 | -0.36 | 0.74 | **4.65** | **8.54** | -0.22 | 0.57 | -0.40 | 0.38 | **3.45** | **9.25** |
|  | **0.5** |  |  | |  |  | **8.10** | | -0.26 | | -0.08 | | **6.32** | | -0.10 | | -0.29 | | **6.08** | |
|  |  |  |  |  |  |  | **6.55** | **9.66** | -0.87 | 0.35 | -0.84 | 0.67 | **4.33** | **8.32** | -0.77 | 0.57 | -0.95 | 0.38 | **3.14** | **9.02** |
|  | **1+** |  |  | |  | |  |  | **-8.36** | | **-8.19** | | -1.78 | | **-8.20** | | **-8.39** | | -2.02 | |
|  |  |  |  |  |  |  |  |  | **-9.84** | **-6.88** | **-9.71** | **-6.66** | -4.20 | 0.64 | **-9.73** | **-6.68** | **-9.89** | **-6.89** | -5.17 | 1.12 |
| **GRN** | **0** |  |  | |  | |  | |  |  | 0.17 | | **6.58** | | 0.16 | | -0.03 | | **6.34** | |
|  |  |  |  |  |  |  |  |  |  |  | -0.39 | 0.74 | **4.58** | **8.58** | -0.26 | 0.58 | -0.46 | 0.40 | **3.44** | **9.24** |
|  | **0.5** |  |  | |  | |  | |  | |  |  | **6.41** | | -0.02 | | 0.00 | | **6.16** | |
|  |  |  |  |  |  |  |  |  |  |  |  |  | **4.43** | **8.38** | -0.66 | 0.63 | -0.85 | 0.44 | **3.25** | **9.08** |
|  | **1+** |  |  | |  | |  | |  | |  | |  |  | **-6.43** | | **-6.61** | | -0.25 | |
|  |  |  |  |  |  |  |  |  |  |  |  |  |  |  | **-8.41** | **-4.44** | **-8.59** | **-4.6** | -3.74 | 3.25 |
| **MAPT** | **0** |  |  | |  | |  | |  | |  | |  | |  |  | -0.19 | | **6.18** | |
|  |  |  |  |  |  |  |  |  |  |  |  |  |  |  |  |  | -0.73 | 0.36 | **3.16** | **9.20** |
|  | **0.5** |  |  | |  | |  | |  | |  | |  | |  | |  |  | **6.37** | |
|  |  |  |  |  |  |  |  |  |  |  |  |  |  |  |  |  |  |  | **3.50** | **9.24** |
|  | **1+** |  |  | |  | |  | |  | |  | |  | |  | |  | |  |  |
|  |  |  |  |  |  |  |  |  |  |  |  |  |  |  |  |  |  |  |  |  |

**i)**

| **Self Care** | | **Healthy controls** | **C9orf72** | | | | | | **GRN** | | | | | | **MAPT** | | | | | |
| --- | --- | --- | --- | --- | --- | --- | --- | --- | --- | --- | --- | --- | --- | --- | --- | --- | --- | --- | --- | --- |
|  |  |  | **0** | | **0.5** | | **1+** | | **0** | | **0.5** | | **1+** | | **0** | | **0.5** | | **1+** | |
| **Healthy controls** | |  | 0.06 | | 0.70 | | **5.19** | | 0.03 | | 0.01 | | **2.53** | | 0.13 | | 0.23 | | **2.73** | |
|  |  |  | -0.19 | 0.31 | -0.07 | 1.47 | **3.86** | **6.52** | -0.15 | 0.22 | -0.29 | 0.31 | **1.14** | **3.93** | -0.38 | 0.65 | -0.20 | 0.65 | **0.22** | **5.24** |
| **C9orf72** | **0** |  |  |  | 0.64 | | **5.13** | | -0.03 | | -0.05 | | **2.47** | | 0.07 | | 0.17 | | **2.67** | |
|  |  |  |  |  | -0.16 | 1.45 | **3.74** | **6.52** | -0.33 | 0.28 | -0.44 | 0.33 | **1.10** | **3.85** | -0.48 | 0.63 | -0.32 | 0.66 | **0.16** | **5.18** |
|  | **0.5** |  |  | |  |  | **4.49** | | -0.67 | | -0.70 | | **1.83** | | -0.57 | | -0.48 | | 2.03 | |
|  |  |  |  |  |  |  | **2.94** | **6.04** | -1.46 | 0.12 | -1.54 | 0.15 | **0.29** | **3.37** | -1.49 | 0.35 | -1.35 | 0.40 | -0.59 | 4.65 |
|  | **1+** |  |  | |  | |  |  | **-5.16** | | **-5.19** | | **-2.66** | | **-5.06** | | **-4.97** | | -2.46 | |
|  |  |  |  |  |  |  |  |  | **-6.51** | **-3.80** | **-6.55** | **-3.83** | **-4.59** | **-0.73** | **-6.53** | **-3.59** | **-6.34** | **-3.60** | -5.20 | 0.28 |
| **GRN** | **0** |  |  | |  | |  | |  |  | -0.03 | | **2.50** | | 0.10 | | 0.19 | | **2.70** | |
|  |  |  |  |  |  |  |  |  |  |  | -0.36 | 0.31 | **1.10** | **3.90** | -0.44 | 0.64 | -0.24 | 0.63 | **0.18** | **5.21** |
|  | **0.5** |  |  | |  | |  | |  | |  |  | **2.53** | | 0.13 | | 0.00 | | **2.73** | |
|  |  |  |  |  |  |  |  |  |  |  |  |  | **1.18** | **3.87** | -0.46 | 0.72 | -0.28 | 0.73 | **0.20** | **5.25** |
|  | **1+** |  |  | |  | |  | |  | |  | |  |  | **-2.40** | | **-2.31** | | 0.20 | |
|  |  |  |  |  |  |  |  |  |  |  |  |  |  |  | **-3.82** | **-0.98** | **-3.79** | **-0.8** | -2.75 | 3.14 |
| **MAPT** | **0** |  |  | |  | |  | |  | |  | |  | |  |  | 0.09 | | 2.60 | |
|  |  |  |  |  |  |  |  |  |  |  |  |  |  |  |  |  | -0.58 | 0.77 | -0.10 | 5.30 |
|  | **0.5** |  |  | |  | |  | |  | |  | |  | |  | |  |  | **2.51** | |
|  |  |  |  |  |  |  |  |  |  |  |  |  |  |  |  |  |  |  | **0.18** | **4.83** |
|  | **1+** |  |  | |  | |  | |  | |  | |  | |  | |  | |  |  |
|  |  |  |  |  |  |  |  |  |  |  |  |  |  |  |  |  |  |  |  |  |

**j)**

| **Memory and Orientation** | | **Healthy controls** | **C9orf72** | | | | | | **GRN** | | | | | | **MAPT** | | | | | |
| --- | --- | --- | --- | --- | --- | --- | --- | --- | --- | --- | --- | --- | --- | --- | --- | --- | --- | --- | --- | --- |
|  |  |  | **0** | | **0.5** | | **1+** | | **0** | | **0.5** | | **1+** | | **0** | | **0.5** | | **1+** | |
| **Healthy controls** | |  | 0.03 | | **1.26** | | **13.50** | | -0.09 | | **2.26** | | **11.05** | | 0.38 | | 1.69 | | **14.07** | |
|  |  |  | -0.58 | 0.63 | **0.07** | **2.44** | **11.82** | **15.19** | -0.56 | 0.37 | **0.83** | **3.70** | **8.61** | **13.49** | -0.34 | 1.11 | -0.27 | 3.65 | **10.59** | **17.54** |
| **C9orf72** | **0** |  |  |  | 1.23 | | **13.48** | | -0.12 | | **2.24** | | **11.02** | | 0.36 | | 1.66 | | **14.04** | |
|  |  |  |  |  | -0.09 | 2.55 | **11.82** | **15.14** | -0.79 | 0.55 | **0.72** | **3.75** | **8.55** | **13.49** | -0.51 | 1.22 | -0.32 | 3.64 | **10.57** | **17.51** |
|  | **0.5** |  |  | |  |  | **12.25** | | **-1.35** | | 1.01 | | **9.79** | | -0.87 | | 0.43 | | **12.81** | |
|  |  |  |  |  |  |  | **10.20** | **14.30** | **-2.56** | **-0.14** | -0.85 | 2.86 | **7.12** | **12.46** | -2.18 | 0.44 | -1.83 | 2.69 | **9.22** | **16.40** |
|  | **1+** |  |  | |  | |  |  | **-13.60** | | **-11.24** | | -2.46 | | **-13.12** | | **-11.82** | | 0.56 | |
|  |  |  |  |  |  |  |  |  | **-15.31** | **-11.89** | **-13.45** | **-9.04** | -5.29 | 0.37 | **-14.97** | **-11.27** | **-14.30** | **-9.33** | -3.13 | 4.25 |
| **GRN** | **0** |  |  | |  | |  | |  |  | **2.36** | | **11.14** | | 0.48 | | 1.78 | | **14.16** | |
|  |  |  |  |  |  |  |  |  |  |  | **0.88** | **3.83** | **8.75** | **13.54** | -0.32 | 1.28 | -0.19 | 3.75 | **10.68** | **17.64** |
|  | **0.5** |  |  | |  | |  | |  | |  |  | **8.79** | | **-1.88** | | 0.00 | | **11.80** | |
|  |  |  |  |  |  |  |  |  |  |  |  |  | **5.83** | **11.74** | **-3.45** | **-0.31** | -2.99 | 1.84 | **8.08** | **15.53** |
|  | **1+** |  |  | |  | |  | |  | |  | |  |  | **-10.66** | | **-9.36** | | 3.02 | |
|  |  |  |  |  |  |  |  |  |  |  |  |  |  |  | **-13.19** | **-8.14** | **-12.46** | **-6.27** | -1.09 | 7.12 |
| **MAPT** | **0** |  |  | |  | |  | |  | |  | |  | |  |  | 1.30 | | **13.68** | |
|  |  |  |  |  |  |  |  |  |  |  |  |  |  |  |  |  | -0.67 | 3.28 | **10.11** | **17.26** |
|  | **0.5** |  |  | |  | |  | |  | |  | |  | |  | |  |  | **12.38** | |
|  |  |  |  |  |  |  |  |  |  |  |  |  |  |  |  |  |  |  | **8.65** | **16.11** |
|  | **1+** |  |  | |  | |  | |  | |  | |  | |  | |  | |  |  |
|  |  |  |  |  |  |  |  |  |  |  |  |  |  |  |  |  |  |  |  |  |

**Table S3: Adjusted mean differences from within-group analysis of each of the ten CBI-R domains for symptomatic mutation carriers (a. *C9orf72,* b. *GRN,* c. *MAPT*) with 95% bootstrapped bias-corrected confidence intervals. Significant values are shown in bold.**

| ***C9orf72*  (CDR = 1+)** | **Stereotypic and Motor Behaviours** | | **Eating Habits** | | **Abnormal Behaviour** | | **Beliefs** | | **Mood** | | **Sleep** | | **Everyday Skills** | | **Self Care** | | **Memory and Orientation** | |  |
| --- | --- | --- | --- | --- | --- | --- | --- | --- | --- | --- | --- | --- | --- | --- | --- | --- | --- | --- | --- |
|  |  |  |  |  |  |  |  |  |  |  |  |  |  |  |  |  |  |  |  |
| **Motivation** | **-10.90** | | **-11.36** | | **-19.23** | | **-39.50** | | **-26.01** | | **-14.81** | | **-8.06** | | **-19.29** | | -2.22 | |  |
|  | **-19.79** | **-2.00** | **-19.58** | **-3.15** | **-27.17** | **-11.29** | **-47.85** | **-31.16** | **-33.48** | **-18.54** | **-24.37** | **-5.26** | **-15.29** | **-0.83** | **-26.97** | **-11.61** | -9.14 | 4.70 |  |
| **Stereotypic and Motor Behaviours** |  | | -0.47 | | **-8.33** | | **-28.61** | | **-15.11** | | -3.92 | | 2.84 | | -8.40 | | **8.68** | |  |
|  |  |  | -7.12 | 6.18 | **-14.92** | **-1.74** | **-36.00** | **-21.21** | **-22.28** | **-7.94** | -12.80 | 4.96 | -6.80 | 12.47 | -18.91 | 2.12 | **1.84** | **15.51** |  |
| **Eating Habits** |  | |  | | **-7.87** | | **-28.14** | | **-14.65** | | -3.45 | | 3.30 | | -7.93 | | **9.14** | |  |
|  |  |  |  |  | **-14.31** | **-1.43** | **-35.40** | **-20.88** | **-22.95** | **-6.35** | -11.31 | 4.41 | -5.49 | 12.09 | -17.75 | 1.89 | **2.15** | **16.13** |  |
| **Abnormal Behaviour** |  | |  | |  | | **-20.27** | | **-6.78** | | 4.42 | | **4.35** | | -0.06 | | **17.01** | |  |
|  |  |  |  |  |  |  | **-26.42** | **-14.13** | **-12.16** | **-1.40** | -3.26 | 12.09 | **2.64** | **19.69** | -9.23 | 9.11 | **11.04** | **22.97** |  |
| **Beliefs** |  | |  | |  | |  | | **13.50** | | **24.69** | | **31.44** | | **20.21** | | **37.28** | |  |
|  |  |  |  |  |  |  |  |  | **8.14** | **18.85** | **18.09** | **31.29** | **23.63** | **39.25** | **11.51** | **28.91** | **31.03** | **43.53** |  |
| **Mood** |  | |  | |  | |  | |  | | **11.19** | | **17.95** | | 6.72 | | **23.79** | |  |
|  |  |  |  |  |  |  |  |  |  |  | **4.27** | **18.12** | **10.01** | **25.88** | -1.56 | 15.00 | **17.32** | **30.25** |  |
| **Sleep** |  | |  | |  | |  | |  | |  | | 6.75 | | -4.48 | | **12.59** | |  |
|  |  |  |  |  |  |  |  |  |  |  |  |  | -2.52 | 16.02 | -13.91 | 4.96 | **4.43** | **20.76** |  |
| **Everyday Skills** |  | |  | |  | |  | |  | |  | |  | | **-11.23** | | 5.84 | |  |
|  |  |  |  |  |  |  |  |  |  |  |  |  |  |  | **-18.24** | **-4.23** | -0.57 | 12.24 |  |
| **Self Care** |  | |  | |  | |  | |  | |  | |  | |  |  | **17.07** | |  |
|  |  |  |  |  |  |  |  |  |  |  |  |  |  |  |  | | **8.59** | **25.55** |  |

| ***GRN*  (CDR = 1+)** | **Stereotypic and Motor Behaviours** | | **Eating Habits** | | **Abnormal Behaviour** | | **Beliefs** | | **Mood** | | **Sleep** | | **Everyday Skills** | | **Self Care** | | **Memory and Orientation** | |  |
| --- | --- | --- | --- | --- | --- | --- | --- | --- | --- | --- | --- | --- | --- | --- | --- | --- | --- | --- | --- |
|  |  |  |  |  |  |  |  |  |  |  |  |  |  |  |  |  |  |  |  |
| **Motivation** | **-26.17** | | **-17.79** | | **-25.82** | | **-41.77** | | **-25.51** | | **-13.67** | | **-14.47** | | **-32.95** | | **-7.69** | |  |
|  | **-34.87** | **-17.47** | **-26.38** | **-9.20** | **-33.65** | **-17.98** | **-50.43** | **-33.12** | **-35.46** | **-15.55** | **-22.70** | **-4.64** | **-22.58** | **-6.36** | **-41.25** | **-24.65** | **-14.60** | **-0.78** |  |
| **Stereotypic and Motor Behaviours** |  | | **8.38** | | 0.35 | | **-15.60** | | 0.66 | | **12.50** | | **11.70** | | -6.78 | | **18.48** | |  |
|  |  |  | **1.28** | **15.48** | -4.45 | 5.16 | **-21.93** | **-9.28** | -5.89 | 7.22 | **2.45** | **22.55** | **2.02** | **21.39** | -15.63 | 2.07 | **10.42** | **26.55** |  |
| **Eating Habits** |  | |  | | **-8.02** | | **-23.98** | | -7.71 | | 4.12 | | 3.32 | | **-15.16** | | 10.11 | |  |
|  |  |  |  |  | **-15.85** | **-0.20** | **-32.21** | **-15.75** | -17.19 | 1.77 | -5.54 | 13.79 | -7.23 | 13.88 | **-24.18** | **-6.13** | -0.08 | 20.29 |  |
| **Abnormal Behaviour** |  | |  | |  | | **-15.96** | | 0.31 | | **12.15** | | **4.29** | | -7.14 | | **18.13** | |  |
|  |  |  |  |  |  |  | **-21.21** | **-10.71** | -5.79 | 6.41 | **2.60** | **21.70** | **2.95** | **19.75** | -14.86 | 0.59 | **12.19** | **24.07** |  |
| **Beliefs** |  | |  | |  | |  | | **16.27** | | **28.10** | | **27.30** | | **8.82** | | **34.09** | |  |
|  |  |  |  |  |  |  |  |  | **11.08** | **21.46** | **18.93** | **37.28** | **17.44** | **37.17** | **1.47** | **16.17** | **26.93** | **41.24** |  |
| **Mood** |  | |  | |  | |  | |  | | **11.84** | | 11.04 | | -7.45 | | **17.82** | |  |
|  |  |  |  |  |  |  |  |  |  |  | **0.99** | **22.68** | -0.03 | 22.10 | -16.99 | 2.10 | **9.36** | **26.28** |  |
| **Sleep** |  | |  | |  | |  | |  | |  | | -0.80 | | **-19.28** | | 5.98 | |  |
|  |  |  |  |  |  |  |  |  |  |  |  |  | -12.05 | 10.46 | **-27.12** | **-11.44** | -3.41 | 15.38 |  |
| **Everyday Skills** |  | |  | |  | |  | |  | |  | |  | | **-18.48** | | 6.78 | |  |
|  |  |  |  |  |  |  |  |  |  |  |  |  |  |  | **-25.83** | **-11.14** | -0.07 | 13.64 |  |
| **Self Care** |  | |  | |  | |  | |  | |  | |  | |  | | **25.27** | |  |
|  |  |  |  |  |  |  |  |  |  |  |  |  |  |  |  |  | **17.73** | **32.80** |  |

| ***MAPT* (CDR = 1+)** | **Stereotypic and Motor Behaviours** | | **Eating Habits** | | **Abnormal Behaviour** | | **Beliefs** | | **Mood** | | **Sleep** | | **Everyday Skills** | | **Self Care** | | **Memory and Orientation** | |  |
| --- | --- | --- | --- | --- | --- | --- | --- | --- | --- | --- | --- | --- | --- | --- | --- | --- | --- | --- | --- |
|  |  |  |  |  |  |  |  |  |  |  |  |  |  |  |  |  |  |  |  |
| **Motivation** | 1.13 | | -5.12 | | **-16.03** | | **-42.22** | | **-22.08** | | **-24.76** | | -12.38 | | **-31.01** | | 4.55 | |  |
|  | -9.46 | 11.73 | -17.89 | 7.65 | **-30.94** | **-1.12** | **-57.15** | **-27.30** | **-38.63** | **-5.54** | **-39.36** | **-10.17** | -26.91 | 2.15 | **-43.15** | **-18.88** | -7.04 | 16.15 |  |
| **Stereotypic and Motor Behaviours** |  | | -6.25 | | **-17.16** | | **-43.35** | | **-23.21** | | **-25.89** | | **-13.51** | | **-32.14** | | 3.42 | |  |
|  |  |  | -14.57 | 2.07 | **-25.45** | **-8.87** | **-54.63** | **-32.08** | **-33.99** | **-12.43** | **-35.05** | **-16.73** | **-26.53** | **-0.49** | **-44.41** | **-19.88** | -7.51 | 14.36 |  |
| **Eating Habits** |  | |  | | -10.91 | | **-37.10** | | **-16.96** | | **-19.64** | | -7.26 | | **-25.89** | | 9.67 | |  |
|  |  |  |  |  | -24.16 | 2.33 | **-51.15** | **-23.05** | **-31.55** | **-2.37** | **-30.85** | **-8.43** | -20.68 | 6.15 | **-38.44** | **-13.34** | -2.51 | 21.85 |  |
| **Abnormal Behaviour** |  | |  | |  | | **-26.19** | | -6.05 | | -8.73 | | 7.01 | | **-14.98** | | **20.59** | |  |
|  |  |  |  |  |  |  | **-34.00** | **-18.39** | -14.85 | 2.75 | -18.19 | 0.73 | -10.09 | 17.39 | **-28.57** | **-1.39** | **9.42** | **31.75** |  |
| **Beliefs** |  | |  | |  | |  | | **20.14** | | **17.46** | | **29.84** | | 11.21 | | **46.78** | |  |
|  |  |  |  |  |  |  |  |  | **13.53** | **26.75** | **10.44** | **24.49** | **17.59** | **42.09** | -1.01 | 23.43 | **37.56** | **55.99** |  |
| **Mood** |  | |  | |  | |  | |  | | -2.68 | | 9.70 | | -8.93 | | **26.64** | |  |
|  |  |  |  |  |  |  |  |  |  |  | -9.67 | 4.32 | -4.60 | 24.00 | -23.85 | 6.00 | **15.28** | **37.99** |  |
| **Sleep** |  | |  | |  | |  | |  | |  | | **12.38** | | -6.25 | | **29.32** | |  |
|  |  |  |  |  |  |  |  |  |  |  |  |  | **0.41** | **24.35** | -19.15 | 6.65 | **20.49** | **38.15** |  |
| **Everyday Skills** |  | |  | |  | |  | |  | |  | |  | | **-18.63** | | **16.93** | |  |
|  |  |  |  |  |  |  |  |  |  |  |  |  |  |  | **-25.57** | **-11.69** | **8.81** | **25.06** |  |
| **Self Care** |  | |  | |  | |  | |  | |  | |  | |  | | **35.57** | |  |
|  |  |  |  |  |  |  |  |  |  |  |  |  |  |  |  |  | **25.81** | **45.32** |  |

**Table S4: Partial correlations between scores in the 10 domains of the CBI-R and volumes of neuroanatomical regions of interest adjusting for disease severity and age (r values and corresponding p values are shown) for the *C9orf72* mutation carriers.**

| **Region of Interest** | **Motivation** | **Stereotypic and Motor Behaviours** | **Eating Habits** | **Abnormal Behaviour** | **Beliefs** | **Mood** | **Sleep** | **Everyday Skills** | **Self Care** | **Memory and Orientation** |
| --- | --- | --- | --- | --- | --- | --- | --- | --- | --- | --- |
| **Left Frontal Lobe** | -0.08 | -0.03 | -0.17 | 0.00 | -0.21 | 0.00 | -0.12 | -0.21 | -0.21 | -0.06 |
|  | 0.319 | 0.648 | 0.024 | 0.965 | 0.005 | 0.951 | 0.111 | 0.006 | 0.006 | 0.438 |
| **Right Frontal Lobe** | -0.09 | 0.03 | -0.14 | 0.03 | -0.17 | -0.04 | -0.09 | -0.24 | -0.26 | -0.04 |
|  | 0.243 | 0.741 | 0.071 | 0.669 | 0.024 | 0.557 | 0.216 | 0.002 | 0.001 | 0.559 |
| **Left Temporal Lobe** | -0.07 | -0.02 | -0.15 | -0.02 | -0.18 | 0.09 | 0.08 | -0.16 | -0.09 | 0.03 |
|  | 0.367 | 0.843 | 0.048 | 0.833 | 0.019 | 0.222 | 0.304 | 0.041 | 0.228 | 0.657 |
| **Right Temporal Lobe** | -0.01 | 0.00 | -0.11 | 0.01 | -0.16 | 0.05 | 0.03 | -0.04 | -0.08 | 0.02 |
|  | 0.895 | 0.970 | 0.163 | 0.899 | 0.039 | 0.486 | 0.732 | 0.598 | 0.283 | 0.811 |
| **Left Parietal Lobe** | -0.09 | -0.06 | -0.13 | 0.04 | -0.18 | -0.04 | -0.05 | -0.22 | -0.11 | -0.08 |
|  | 0.231 | 0.430 | 0.094 | 0.597 | 0.017 | 0.578 | 0.481 | 0.003 | -0.106 | 0.310 |
| **Right Parietal Lobe** | -0.05 | -0.04 | -0.09 | 0.01 | -0.14 | -0.04 | -0.07 | -0.23 | -0.12 | -0.05 |
|  | 0.526 | 0.641 | 0.251 | 0.889 | 0.061 | 0.629 | 0.392 | 0.002 | 0.106 | 0.531 |
| **Left Insula** | -0.07 | -0.02 | -0.22 | 0.01 | -0.10 | 0.05 | -0.05 | -0.19 | -0.17 | 0.02 |
|  | 0.366 | 0.763 | 0.004 | 0.903 | 0.197 | 0.534 | 0.548 | 0.014 | 0.029 | 0.749 |
| **Right Insula** | -0.05 | 0.05 | -0.20 | 0.01 | -0.08 | 0.06 | -0.05 | -0.13 | -0.13 | 0.02 |
|  | 0.514 | 0.542 | 0.009 | 0.941 | 0.292 | 0.422 | 0.524 | 0.077 | 0.097 | 0.756 |
| **Left Hippocampus** | -0.11 | -0.14 | -0.18 | -0.16 | -0.06 | -0.09 | -0.09 | -0.11 | -0.06 | -0.22 |
|  | 0.164 | 0.059 | 0.019 | 0.030 | 0.407 | 0.255 | 0.232 | 0.16 | 0.433 | 0.003 |
| **Right Hippocampus** | -0.07 | -0.21 | -0.08 | -0.19 | -0.06 | -0.10 | -0.06 | -0.07 | -0.05 | -0.21 |
|  | 0.371 | 0.006 | 0.295 | 0.010 | 0.409 | 0.179 | 0.432 | 0.359 | 0.505 | 0.005 |
| **Left Amygdala** | -0.01 | -0.14 | -0.04 | -0.07 | -0.08 | 0.16 | 0.04 | -0.09 | -0.01 | -0.12 |
|  | 0.864 | 0.072 | 0.597 | 0.348 | 0.271 | 0.034 | 0.587 | 0.249 | 0.947 | 0.120 |
| **Right Amygdala** | -0.07 | -0.18 | -0.01 | -0.13 | -0.07 | 0.12 | -0.05 | -0.08 | 0.04 | -0.16 |
|  | 0.371 | 0.015 | 0.942 | 0.086 | 0.341 | 0.123 | 0.552 | 0.273 | 0.625 | 0.032 |
| **Left Thalamus** | -0.02 | -0.15 | -0.09 | -0.08 | -0.15 | 0.06 | -0.01 | -0.02 | 0.05 | -0.26 |
|  | 0.814 | 0.042 | 0.223 | 0.272 | 0.040 | 0.456 | 0.874 | 0.812 | 0.531 | 0.001 |
| **Right Thalamus** | -0.02 | -0.17 | -0.07 | -0.11 | -0.13 | 0.01 | -0.02 | -0.01 | 0.04 | -0.23 |
|  | 0.757 | 0.022 | 0.383 | 0.131 | 0.083 | 0.911 | 0.771 | 0.934 | 0.577 | 0.003 |
| **Left Striatum** | -0.05 | -0.13 | -0.06 | -0.08 | -0.16 | 0.12 | 0.06 | -0.13 | 0.06 | -0.07 |
|  | 0.545 | 0.096 | 0.420 | 0.285 | 0.037 | 0.120 | 0.444 | 0.099 | 0.423 | 0.390 |
| **Right Striatum** | -0.02 | -0.07 | -0.07 | -0.03 | -0.17 | 0.13 | 0.06 | -0.12 | 0.06 | -0.03 |
|  | 0.826 | 0.382 | 0.387 | 0.674 | 0.029 | 0.088 | 0.418 | 0.121 | 0.463 | 0.662 |

**Table S5: Partial correlations between scores in the 10 domains of the CBI-R and volumes of neuroanatomical regions of interest adjusting for disease severity and age (r values and corresponding p values are shown) for the *GRN* mutation carriers.**

| **Region of Interest** | **Motivation** | **Stereotypic and Motor Behaviours** | **Eating Habits** | **Abnormal Behaviour** | **Beliefs** | **Mood** | **Sleep** | **Everyday Skills** | **Self Care** | **Memory and Orientation** |
| --- | --- | --- | --- | --- | --- | --- | --- | --- | --- | --- |
| **Left Frontal Lobe** | -0.06 | 0.05 | -0.08 | 0.10 | 0.07 | 0.04 | -0.03 | -0.15 | -0.07 | -0.08 |
|  | 0.391 | 0.494 | 0.292 | 0.170 | 0.345 | 0.565 | 0.648 | 0.050 | 0.345 | 0.269 |
| **Right Frontal Lobe** | -0.08 | 0.03 | -0.16 | 0.04 | -0.07 | 0.04 | -0.06 | -0.04 | -0.20 | -0.05 |
|  | 0.301 | 0.721 | 0.029 | 0.605 | 0.367 | 0.566 | 0.429 | 0.620 | 0.008 | 0.503 |
| **Left Temporal Lobe** | -0.08 | 0.01 | 0.00 | -0.07 | 0.05 | -0.14 | -0.12 | -0.11 | -0.12 | -0.12 |
|  | 0.275 | 0.892 | 0.974 | 0.365 | 0.539 | 0.067 | 0.120 | 0.134 | 0.120 | 0.105 |
| **Right Temporal Lobe** | -0.10 | -0.04 | -0.19 | -0.10 | -0.08 | -0.10 | -0.13 | 0.03 | -0.18 | -0.06 |
|  | 0.195 | 0.616 | 0.012 | 0.190 | 0.281 | 0.172 | 0.078 | 0.696 | 0.014 | 0.403 |
| **Left Parietal Lobe** | -0.14 | -0.06 | -0.03 | -0.04 | 0.02 | -0.07 | -0.04 | -0.14 | -0.13 | -0.12 |
|  | 0.065 | 0.418 | 0.692 | 0.597 | 0.807 | 0.329 | 0.565 | 0.068 | 0.091 | 0.113 |
| **Right Parietal Lobe** | -0.09 | 0.03 | -0.10 | -0.01 | -0.09 | -0.03 | -0.07 | -0.05 | -0.20 | -0.08 |
|  | 0.241 | 0.665 | 0.204 | 0.856 | 0.237 | 0.690 | 0.365 | 0.526 | 0.007 | 0.282 |
| **Left Insula** | -0.05 | -0.03 | -0.17 | -0.01 | 0.03 | -0.08 | -0.05 | -0.21 | -0.10 | -0.05 |
|  | 0.529 | 0.695 | 0.020 | 0.879 | 0.674 | 0.316 | 0.502 | 0.005 | 0.167 | 0.512 |
| **Right Insula** | -0.06 | -0.11 | -0.28 | -0.10 | -0.03 | -0.04 | -0.07 | -0.16 | -0.19 | -0.14 |
|  | 0.404 | 0.154 | <0.001 | 0.180 | 0.723 | 0.588 | 0.326 | 0.036 | 0.012 | 0.065 |
| **Left Hippocampus** | -0.09 | -0.19 | -0.08 | -0.14 | -0.10 | -0.18 | -0.16 | -0.09 | -0.12 | -0.18 |
|  | 0.248 | 0.009 | 0.267 | 0.053 | 0.198 | 0.015 | 0.029 | 0.214 | 0.110 | a0 |
| **Right Hippocampus** | -0.05 | -0.26 | -0.08 | -0.21 | -0.21 | -0.17 | -0.14 | -0.08 | -0.18 | -0.15 |
|  | 0.530 | 0.001 | 0.302 | 0.004 | 0.005 | 0.026 | 0.065 | 0.262 | 0.014 | 0.046 |
| **Left Amygdala** | -0.03 | -0.15 | -0.13 | -0.04 | -0.08 | -0.10 | -0.21 | -0.15 | -0.19 | -0.12 |
|  | 0.735 | 0.040 | 0.087 | 0.632 | 0.294 | 0.194 | 0.005 | 0.040 | 0.009 | 0.106 |
| **Right Amygdala** | -0.06 | -0.19 | -0.17 | -0.04 | -0.07 | -0.07 | -0.19 | -0.08 | -0.16 | -0.14 |
|  | 0.446 | 0.013 | 0.023 | 0.625 | 0.319 | 0.374 | 0.011 | 0.309 | 0.029 | 0.067 |
| **Left Thalamus** | 0.00 | 0.01 | 0.00 | -0.07 | -0.02 | -0.13 | -0.13 | -0.07 | -0.07 | 0.00 |
|  | 0.974 | 0.929 | 0.965 | 0.356 | 0.802 | 0.076 | 0.074 | 0.325 | 0.338 | 0.978 |
| **Right Thalamus** | -0.04 | -0.04 | -0.11 | -0.08 | -0.19 | -0.06 | -0.14 | 0.00 | -0.19 | -0.03 |
|  | 0.631 | 0.583 | 0.130 | 0.301 | 0.010 | 0.405 | 0.067 | 0.950 | 0.010 | 0.655 |
| **Left Striatum** | -0.04 | -0.02 | -0.03 | 0.02 | 0.00 | -0.01 | -0.08 | -0.19 | -0.07 | -0.13 |
|  | 0.617 | 0.792 | 0.651 | 0.748 | 0.998 | 0.856 | 0.302 | 0.009 | 0.352 | 0.085 |
| **Right Striatum** | -0.07 | -0.05 | -0.13 | -0.01 | -0.02 | 0.00 | -0.11 | -0.15 | -0.12 | -0.15 |
|  | 0.382 | 0.514 | 0.095 | 0.855 | 0.795 | 0.948 | 0.156 | 0.049 | 0.106 | 0.046 |

**Table S6: Partial correlations between scores in the 10 domains of the CBI-R and volumes of neuroanatomical regions of interest adjusting for disease severity and age (r values and corresponding p values are shown) for the *MAPT* mutation carriers.**

| **Region of Interest** | **Motivation** | **Stereotypic and Motor Behaviours** | **Eating Habits** | **Abnormal Behaviour** | **Beliefs** | **Mood** | **Sleep** | **Everyday Skills** | **Self Care** | **Memory and Orientation** |
| --- | --- | --- | --- | --- | --- | --- | --- | --- | --- | --- |
| **Left Frontal Lobe** | -0.16 | 0.00 | -0.26 | -0.20 | -0.13 | -0.16 | -0.23 | -0.25 | -0.21 | -0.06 |
|  | 0.203 | 0.983 | 0.037 | 0.113 | 0.285 | 0.200 | 0.059 | 0.039 | 0.081 | 0.646 |
| **Right Frontal Lobe** | -0.17 | 0.00 | -0.31 | -0.10 | -0.11 | -0.21 | -0.23 | -0.28 | -0.22 | -0.09 |
|  | 0.160 | 0.995 | 0.011 | 0.410 | 0.385 | 0.091 | 0.058 | 0.022 | 0.068 | 0.483 |
| **Left Temporal Lobe** | -0.17 | -0.19 | -0.22 | -0.22 | 0.04 | -0.17 | -0.12 | -0.17 | -0.11 | -0.13 |
|  | 0.176 | 0.128 | 0.078 | 0.073 | 0.758 | 0.174 | 0.318 | 0.159 | 0.376 | 0.292 |
| **Right Temporal Lobe** | -0.20 | -0.16 | -0.19 | -0.11 | 0.02 | -0.21 | -0.09 | -0.25 | -0.12 | -0.07 |
|  | 0.113 | 0.207 | 0.116 | 0.385 | 0.863 | 0.090 | 0.462 | 0.042 | 0.322 | 0.597 |
| **Left Parietal Lobe** | 0.01 | 0.06 | -0.17 | 0.00 | 0.02 | -0.06 | -0.08 | -0.07 | -0.18 | -0.04 |
|  | 0.943 | 0.638 | 0.165 | 0.970 | 0.883 | 0.606 | 0.510 | 0.560 | 0.145 | 0.766 |
| **Right Parietal Lobe** | 0.06 | 0.12 | -0.09 | 0.03 | 0.01 | -0.10 | -0.09 | 0.03 | -0.23 | -0.01 |
|  | 0.630 | 0.352 | 0.475 | 0.804 | 0.940 | 0.444 | 0.479 | 0.781 | 0.066 | 0.941 |
| **Left Insula** | -0.09 | -0.16 | -0.12 | -0.33 | 0.10 | -0.14 | -0.15 | -0.12 | -0.16 | -0.12 |
|  | 0.456 | 0.204 | 0.349 | 0.007 | 0.410 | 0.271 | 0.219 | 0.339 | 0.204 | 0.328 |
| **Right Insula** | -0.06 | -0.05 | -0.13 | -0.13 | 0.07 | -0.15 | -0.18 | -0.15 | -0.18 | -0.09 |
|  | 0.641 | 0.710 | 0.282 | 0.300 | 0.594 | 0.218 | 0.143 | 0.241 | 0.155 | 0.471 |
| **Left Hippocampus** | -0.04 | -0.18 | -0.14 | -0.20 | -0.02 | -0.06 | -0.04 | -0.19 | -0.01 | -0.27 |
|  | 0.736 | 0.154 | 0.242 | 0.103 | 0.879 | 0.658 | 0.765 | 0.128 | 0.938 | 0.029 |
| **Right Hippocampus** | -0.12 | -0.22 | -0.29 | -0.11 | -0.05 | -0.18 | -0.18 | -0.32 | -0.15 | -0.32 |
|  | 0.339 | 0.078 | 0.017 | 0.365 | 0.715 | 0.151 | 0.153 | 0.009 | 0.231 | 0.008 |
| **Left Amygdala** | -0.07 | -0.10 | -0.04 | -0.11 | -0.08 | -0.04 | -0.01 | -0.11 | -0.03 | -0.20 |
|  | 0.578 | 0.403 | 0.774 | 0.357 | 0.517 | 0.774 | 0.911 | 0.395 | 0.792 | 0.105 |
| **Right Amygdala** | -0.11 | -0.11 | -0.05 | -0.06 | -0.12 | -0.14 | -0.15 | -0.08 | -0.20 | -0.23 |
|  | 0.362 | 0.363 | 0.673 | 0.632 | 0.346 | 0.251 | 0.213 | 0.496 | 0.110 | 0.058 |
| **Left Thalamus** | -0.09 | -0.19 | -0.23 | -0.21 | -0.12 | -0.05 | -0.10 | -0.27 | 0.08 | -0.13 |
|  | 0.449 | 0.123 | 0.065 | 0.093 | 0.353 | 0.675 | 0.410 | 0.029 | 0.521 | 0.293 |
| **Right Thalamus** | -0.04 | -0.05 | -0.22 | -0.09 | -0.10 | -0.08 | -0.11 | -0.23 | 0.02 | -0.04 |
|  | 0.762 | 0.698 | 0.077 | 0.453 | 0.409 | 0.538 | 0.360 | 0.058 | 0.869 | 0.718 |
| **Left Striatum** | -0.18 | -0.13 | -0.22 | -0.19 | -0.02 | -0.11 | -0.08 | -0.24 | -0.03 | -0.09 |
|  | 0.156 | 0.283 | 0.077 | 0.123 | 0.894 | 0.371 | 0.541 | 0.047 | 0.792 | 0.463 |
| **Right Striatum** | -0.15 | -0.11 | -0.21 | -0.16 | -0.01 | -0.16 | -0.07 | -0.20 | -0.09 | -0.05 |
|  | 0.212 | 0.378 | 0.091 | 0.183 | 0.940 | 0.185 | 0.554 | 0.113 | 0.463 | 0.705 |

**Figure S1: Correlations between CBI-R total scores and i) on the left, CDR plus NACC FTLD sum of boxes scores [*C9orf72* (r = 0.78, *p* < 0.001), *GRN* (r = 0.82, *p* < 0.001) and *MAPT* (r = 0.60, *p* < 0.001)], and ii) on the right, FRS scores [C9orf72 (r = - 0.92, p < 0.001), GRN (r = -0.88, p < 0.001) and MAPT (r = -0.88, p < 0.001)].**

**Figure S2: CBI-R individual domain scores (as a percentage) in each of the ten domains in all symptomatic mutation carrier groups: a. *C9orf72,* b. *GRN,* c. *MAPT.* The error bars represent standard error of the mean.**
